# Pain Science Education, Stress Management, and Cognition-Targeted Exercise Therapy in Chronic Whiplash Disorders: A Randomized Clinical Trial

**DOI:** 10.1001/jamanetworkopen.2025.26674

**Published:** 2025-08-12

**Authors:** Anneleen Malfliet, Dorine Lenoir, Carlos Murillo, Eva Huysmans, Barbara Cagnie, Mira Meeus, Ward Willaert, Kelly Ickmans, Lieven Danneels, Jente Bontinck, Jo Nijs, Iris Coppieters

**Affiliations:** 1 Pain in Motion Research Group, Department of Physiotherapy, Human Physiology and Anatomy, Faculty of Physical Education & Physiotherapy, Vrije Universiteit Brussel, Brussels, Belgium; 2 Research Foundation–Flanders, Brussels, Belgium; 3 Chronic Pain Rehabilitation, Department of Physical Medicine and Physiotherapy, University Hospital Brussels, Brussels, Belgium; 4 Pain in Motion International Research Consortium; 5 Department of Rehabilitation Sciences, Faculty of Medicine and Health Sciences, Ghent University, Ghent, Belgium; 6 Department of Rehabilitation Sciences and Physiotherapy, Faculty of Medicine and Health Sciences, University of Antwerp, Campus Drie Eiken, Wilrijk, Belgium; 7 Department of Health and Rehabilitation, Unit of Physiotherapy, Institute of Neuroscience and Physiology, Sahlgrenska Academy, University of Gothenburg, Gothenburg, Sweden; 8 Experimental Health Psychology Research Group, Faculty of Psychology and Neuroscience, Maastricht University, Maastricht, the Netherlands; 9 The Laboratory for Brain-Gut Axis Studies, Translational Research Center for Gastrointestinal Disorders, KU Leuven, Leuven, Belgium

## Abstract

**Importance:**

Chronic whiplash-associated disorders (cWAD) pose significant personal and societal burden, with limited evidence-based management options.

**Objective:**

To compare the efficacy of the modern pain neuroscience approach (MPNA) with usual care physiotherapy (UC) in cWAD.

**Design, Setting, and Participants:**

This multicenter randomized clinical trial with long-term follow-up was organized at 2 university hospitals and 1 regional hospital in Belgium. Participants included patients with cWAD aged 18 to 65 years. Data collection occurred between August 2017 and July 2022. Final analyses were performed from June to August 2024.

**Interventions:**

Participants were randomized into MPNA or UC and received 18 sessions during 16 weeks. MPNA included pain neuroscience education, stress management, and cognition-targeted, time-contingent exercise therapy. UC included biomedically focused education and symptom-contingent exercise therapy.

**Main Outcomes and Measures:**

The primary end point was pain-related disability (ie, Neck Disability Index) at the 6-month follow-up. Exploratory secondary outcomes included pain, pain cognitions, quality of life, physical functioning, and cost utility, with assessments until 12 months after the intervention.

**Results:**

A total of 120 participants (mean [SD] age, 41.4 [11.3] years; 89 [74.2%] women) were included. Mean neck pain–related disability decreased by 5.74 points (33.3%) in the MPNA group and 3.22 points (15.8%) in the UC group. There was no significant difference at 6 months (mean group difference [MGD], 2.38 [95% CI, −0.12 to 4.89] points; *P* = .15; Cohen *d* = 0.646), but significant differences favored MPNA immediately after treatment (MGD, 3.55 [95% CI, 1.12-6.26] points; *P* = .006; Cohen *d* = 0.999) and at 12-month follow-up (MGD, 3.54 [95% CI, 0.81-6.28] points; *P* = .02; Cohen *d* = 0.947). On an explorative basis, MPNA consistently outperformed UC in improving fear-avoidance behavior (Cohen *d* range, 0.906-1.112) and self-reported central sensitization symptoms (Cohen *d* range, 1.158-1.834) and was cost-effective, saving US $854.01 with an increase of 0.035 quality-adjusted life-years. No group differences were found for quality of life, (experimental) pain, and pain catastrophizing. However, 14 (23.3%) of the MPNA group was free of pain (ie, 0 or 1 of 10 in pain intensity score) directly after treatment vs only 3 (5.0%) in the UC group.

**Conclusions and Relevance:**

In this randomized clinical trial, no significant differences were observed between groups for the primary outcome at 6 months. MPNA was more effective than UC in reducing neck pain–related disability immediately after treatment and at 12-month follow-up, with additional benefits in pain-related anxiety, self-reported central sensitization symptoms, and cost-effectiveness. Overall, MPNA shows promise for managing cWAD.

**Trial Registration:**

ClinicalTrials.gov Identifier: NCT03239938

## Key Points

**Question:**

Is the modern pain neuroscience approach (MPNA) more effective than usual care physiotherapy in improving disability, pain, function, quality of life, and cost utility in chronic whiplash-associated disorders (cWAD)?

**Findings:**

This randomized clinical trial of 120 participants found a negative result for the prespecified primary outcome (ie, disability at 6-month follow-up). However, secondary analysis showed that MPNA was cost-effective and reduced disability immediately after treatment and at 12-month follow-up and better addressed fear avoidance and central sensitization symptoms.

**Meaning:**

MPNA appears promising for cWAD management in the absence of strong guidelines, although further research is needed to optimize its delivery and identify responders.

## Teaser

This randomized clinical trial evaluates whether pain neuroscience education combined with stress management and cognition-targeted exercise therapy is superior to usual care in chronic whiplash-associated disorders.

## Introduction

Whiplash-associated disorders (WAD) include a range of symptoms resulting from indirect neck trauma, often caused by acceleration-deceleration mechanisms.^1^ While many individuals recover, approximately half will continue to experience ongoing pain and disability 1 year later (ie, chronic WAD [cWAD]).^2,3^ Therefore, neck trauma is acknowledged as a significant global health issue, with the economic burden in Europe alone reaching US $22 billion annually.^4^ These costs are largely attributed to ongoing treatment and reduced work productivity.^4^

As functional disabilities in cWAD depend on individual recovery prerequisites and biomechanical preconditions, its management calls for individuality.^5,6^ Additionally, research indicates that active interventions are generally most cost-effective.^7,8^ However, managing cWAD remains challenging, as evidence-based guidance is currently limited due to inconsistent outcomes.^9,10,11,12^ This may be partly due to an inadequate integration of advances in cWAD research, particularly concerning its complex pathophysiology—including altered brain structure and function, impaired exercise-induced hypoalgesia, dysfunctional stress regulation, and psychological factors such as pain-related anxiety^13,14,15,16,17^—which collectively complicate cWAD symptomatology.^13,14,15,16^

A previous study that included members of our group^18^ sought to address the underlying psychobiological chronic pain mechanisms by integrating pain science education with cognition-targeted exercise therapy in a broader sample of patients with nonspecific spinal pain. This approach targeted pain modulatory mechanisms and cognitive factors associated with pain.^19^ Results demonstrated superiority over usual care (UC) with improvements in pain, disability, mental and physical functioning, and pain cognitions.^18^ However, with only 10% of the sample having cWAD, generalizability to this population was limited. This is particularly challenging, as cWAD is often positioned at the more severe end of the nociplastic pain continuum, whereas nontraumatic neck pain and nonspecific low back pain typically fall at the less severe end.^9^ Moreover, many individuals with cWAD experience mild to severe posttraumatic stress and a blunted stress response, which are known risk factors for poor recovery.^20,21,22,23^ Addressing dysregulation of the stress response system by integrating stress management into treatment may be beneficial for coping, pain-related disability, pain intensity, and quality of life.^20,21,22,23^ As such, a comprehensive multimodal treatment, including pain neuroscience education, stress management, and cognition-targeted exercise therapy, is suggested, but this regimen currently lacks evidence.^23^

Despite this limitation, existing evidence cautiously suggests that pain neuroscience education, stress management, and exercise therapy, when applied separately, may benefit individuals with cWAD.^1,9,20,21,24^ Therefore, combining these interventions within a structured neuroscientific framework holds considerable potential for this population. To address this gap, we performed the first sufficiently powered multicenter randomized clinical trial (RCT), to our knowledge, with long-term follow-up to evaluate whether pain neuroscience education combined with stress management and cognition-targeted exercise therapy is superior to UC (ie, biomedically focused education and symptom-contingent exercise therapy) in cWAD. We hypothesized that this combined approach would be more effective to improve pain-related disability, pain, health care costs, function, and pain cognitions.

## Methods

### Design and Blinding

This multicenter, randomized, 2-arm superiority clinical trial with long-term follow-up was approved by the ethics boards of 2 university hospitals (Ghent University Hospital and University Hospital Brussels) and 1 local hospital (AZ Rivierenland, campus Bornem) in Belgium. Assessors (W.W. and D.L.) and the statistician (C.M.) were blinded to the allocation, whereas participants were not blinded to the intervention but were unaware of the study hypothesis. Written informed consent was obtained prior to any study related procedure. This study follows the Consolidated Standards of Reporting Trials (CONSORT) guidelines. Recruitment and data collection occurred between August 2017 and July 2022. The study protocol can be found in Supplement 1 and elsewhere.^25^

### Study Population

Participants with cWAD (18-65 years of age) were recruited via the participating universities and university hospitals, through social media, primary care practices, pharmacies, patient support groups, and advertisements in radio, newspapers, magazines, symposia, health insurance companies, and district health centers. Details on eligibility criteria are included in eTable 1 in Supplement 2 and elsewhere.^25^

### Randomization

Stratified permuted block randomization (1:1) was computer generated at the Ghent University Biostatistics Unit by an independent investigator. Separate randomization lists were prepared for each treatment center (n = 3). Sequentially numbered, opaque, sealed envelopes held group assignments. An independent researcher (I.C.) uninvolved in recruitment, assessments, or treatment added participants’ initials before opening each envelope to ensure allocation concealment.

### Trial Monitoring

Treatment adherence was assessed using the completed vs prescribed therapy sessions ratio. Concomitant care and adverse events were monitored using self-report at each time point. Participant retention was supported with active reminders and gift vouchers on trial completion.

### Outcome Measures

Data collection was performed at baseline, immediately after treatment, and at the 6- and 12-month follow-ups (Trial Protocol in Supplement 1).^25^ The primary clinical outcome was pain-related disability, assessed using the valid and reliable Neck Disability Index (NDI), with a score ranging from 0 to 50 (cutoff score indicating disability, ≥15 of 50)^26,27,28^ and a minimal clinical important difference (MCID) of 3.5 points.^29^ The primary end point occurred at the 6-month follow-up.

Explorative, secondary pain-related outcomes included pain intensity using an 11-point numeric rating scale (MCID, 1.5 points)^30,31^; pain frequency; pain location and extent; and self-reported symptoms of central sensitization, assessed via the Central Sensitization Inventory (cutoff score indicative of central sensitization symptoms, ≥40 of 100.)^32,33^ Experimental pain was assessed using quantitative sensory testing, including electrical detection and pain thresholds, as well as the evaluation of endogenous pain modulation.^34,35,36,37^ Details of this protocol are available in eMethods 1 in Supplement 2. Other explorative secondary outcomes included health-related quality of life (ie, 36-Item Short Form Health Survey [SF-36]),^38^ physical functioning (ie, Patient Specific Complaints Questionnaire),^39^ pain catastrophizing (ie, Pain Catastrophizing Scale),^40,41,42^ symptoms of posttraumatic stress (ie, Impact of Event Scale–Revised),^43^ illness perceptions (ie, Illness Perception Questionnaire–Revised),^44^ and pain-related fear and anxiety (ie, Pain Anxiety Symptoms Scale, short version [PASS-20]).^45,46^

For the cost-utility analysis, health effects were determined based on the SF-36 from which utilities and quality-adjusted life-years (QALYs) were calculated. For costs, a societal perspective was applied considering both health care and productivity loss–related costs. All details on this analysis are available in eMethods 2 in Supplement 2.

### Intervention

Both groups received 18 therapy sessions during 16 weeks. All sessions were one-on-one (except for 1 group session), using principles of person-centered care and applying guidance toward self-management, and were delivered by expert-trained physical therapists. Therapists involved in one treatment arm were not involved in the other treatment arm and vice versa. Details on the training of these therapists can be found in eTable 2 in Supplement 2. The modern pain neuroscience approach (MPNA) consisted of 3 pain neuroscience education sessions (totaling 2 hours of education) combined with 15 sessions of cognition-targeted, time-contingent exercise therapy and stress management (totaling 7.5 hours).^47^ Usual care physiotherapy (UC) included 3 biomedically focused neck school education sessions combined with 15 sessions of symptom-contingent exercise therapy (totaling 2 and 7.5 hours, respectively). The key treatment difference is that MPNA incorporates a reconceptualization of pain focusing on the brain’s central role, along with stress management and exercise therapy not guided by pain. These elements were not included in UC. Full details are given in the trial protocol (Supplement 1) and elsewhere.^25,47^

### Statistical Analysis

Data were analyzed from June to August 2024. The sample size (n = 120) was calculated using G*Power software, version 3.1.9.2 (Universität Düsseldorf), for the primary end point based on a similar RCT^18^ (F test, partial η^2^ = 0.032; effect size = 0.18; α = .05; power = 0.80), accounting for 25% loss to follow-up.^25^ Statistical analyses were performed in R package Ime4, version 1.1-35.4, (R Program for Statistical Computing), using an intention-to-treat approach, with missing data handled under the assumption that they were missing at random. Effects for NDI at the primary end point and the other time points were analyzed using linear mixed models with random intercept and restricted maximum likelihood. The model (adjusted for age and sex) included fixed effects for treatment allocation (experimental or control group), time, and treatment by time interaction. Given the involvement of 3 treatment centers, analyses were performed both with and without center as random effect, allowing comparison of the model’s fit. Mean group differences (95% CI) with *P* values (significance level, 2-sided α = .05) and effect sizes using Cohen *d* for between group comparisons and partial η^2^ for the interaction effects are reported. The same analysis evaluated the explorative, secondary outcomes at the different time points. For pain intensity, the percentage of individuals deemed free of pain (ie, 0 or 1 of 10 in pain intensity directly after treatment) were calculated for both groups separately. A sensitivity analysis, adjusting for baseline NDI levels, was performed for the primary outcome. The cost-utility analysis was performed using Excel, version 2024 (Microsoft Corporation), and SPSS, version 29 (IBM Corporation), and consisted of a base case and several scenario analyses, including probabilistic sensitivity analyses eMethods 2 in Supplement 2).

## Results

### Flow of the Participants Through the Study

In this randomized clinical trial, 120 participants (mean [SD] age, 41.4 [11.3] years; 31 [25.8%] men and 89 [74.2%] women) were included. Table 1 and Table 2 present the participants’ demographic and baseline characteristics. Full details on the study flow are presented in the Figure. A significant proportion of screened individuals were ineligible for participation, with primary reasons being language barriers, pregnancy, and low NDI scores. This high ineligibility rate was likely driven by the study’s extensive and broad recruitment strategy rather than overly restrictive sample criteria. Participant recruitment took longer than expected, primarily due to COVID-19 restrictions. eTable 6 in Supplement 2 reports details on missing data; eTable 8 in Supplement 2 includes baseline characteristics of dropouts vs study completers.

**Table 1.** Demographic Variables and Baseline Characteristics of the Participating Patients With cWAD

Characteristic	Participant group, mean (SD) [range]	
	UC (n = 60)	MPNA (n = 60)
Demographic		
Age, y	42.4 (11.8) [19.0-64.0]	40.5 (10.8) [23.0-62.0]
Body mass index^a^	25.1 (3.58) [18.8-32.2]	24.8 (4.65) [16.7-36.8]
Pain-related variables		
Neck Disability Index score^b^	19.9 (5.13) [7.0-35.0]	18.6 (4.9) [8.0-32.0]
Days with pain last month	5.8 (1.61) [1.0-7.0]	5.9 (1.5) [2.0-7.0]
Mean pain previous week, Numeric Pain Rating Scale score^c^	5.4 (1.7) [1.0-8.0]	5.3 (1.8) [1.0-9.0]
Patient Specific Complaints Questionnaire score^d^	19.5 (5.4) [0-28.0]	19.8 (4.7) [7.0-29.0]
SF-36 Physical Functioning score, %^e^	33.4 (6.9) [16.1-49.8]	32.8 (7.9) [17.5-50.6]
SF-36 Mental Functioning score, %^e^	48.3 (14.2) [19.0-77.5]	51.6 (12.2) [21.9-74.7]
Pain Catastrophizing Scale score^f^	24.5 (10.9) [4.0-47.0]	23.5 (11.7) [1.0-49.0]
Pain-related fear, Pain Anxiety Symptoms Scale, short form, score^g^	35.8 (18.9) [7.0-89.0]	31.6 (19.0) [4.0-94.0]
Central Sensitization Inventory^h^	49.0 (13.7) [17.0-83.0]	44.5 (12.6) [15.0-73.0]
Impact of Event Scale–Revised^i^	20.5 (14.7) [3.0-65.0]	16.5 (16.0) [0-61]
Illness Perception Questionnaire–Revised^j^	130.0 (12.4) [106.0-157.0]	128.0 (13.0) [104.0-159.0]

Abbreviations: cWAD, chronic whiplash-associated disorder; MPNA, modern pain neuroscience approach; SF-36, 33-Item Short Form Health Survey; UC, usual care.

^a^ Calculcated as the weight in kilograms divided by the square of the height in meters.

^b^ Scores range from 0 to 50, with scores of 15 or greater indicating disability.

^c^ Scores range from 0 to 10, with higher scores indicating higher pain intensity.

^d^ Scores range from 0 to 30, with higher scores indicating more inconvenience.

^e^ Scores range from 0 to 100%, with higher scores indicating better functioning.

^f^ Scores range from 0 to 52, with higher scores indicating more catastrophizing.

^g^ Scores range from 0 to 100, with higher scores indicating higher pain anxiety symptoms.

^h^ Scores range from 0 to 100, with higher scores indicating more self-reported symptoms of central sensitization.

^i^ Scores range from 0 to 88, with higher scores indicating more distress.

^j^ Scores range from 0 to 204, with higher scores indicating stronger beliefs or perceptions.

**Table 2.** Demographic Variables of Participating Patients With cWAD

Variable	Participant group, No. (%)	
	UC (n = 60)	MPNA (n = 60)
Sex		
Female	47 (78.3)	42 (70.0)
Male	13 (21.7)	18 (30.0)
Educational level		
Primary	3 (5.0)	0 (0.0)
Secondary	19 (31.7)	25 (41.7)
Vocational	26 (43.3)	25 (41.7)
Bachelor’s degree, master’s degree, or PhD	12 (20.0)	10 (16.7)
Marital status		
Single	19 (31.7)	13 (21.7)
Married	22 (36.7)	23 (38.3)
Cohabitation	14 (23.3)	11 (18.3)
Divorced	4 (6.7)	10 (16.7)
Widowed	1 (1.7)	3 (5.0)
Employment status ^a^		
Student	5 (8.3)	0
Unemployed	2 (3.3)	3 (5.0)
Self-employed	0	3 (5.0)
Employed	38 (63.3)	48 (80.0)
Incapacitated	12 (20.0)	4 (6.7)
Retired	3 (5.0)	1 (1.7)
Annual income, US $^b^		
<11 368 (<€10 000)	0	1 (1.7)
11 368 to <22 736 (€10 000-20 000)	7 (11.7)	12 (20.0)
22 736 to <45 473 (€20 000-€40 000)	33 (55.0)	19 (31.7)
45 473 to <68 209 (€40 000-€60 000)	8 (13.3)	22 (36.7)
≥68 209 (>€60 000)	5 (8.3)	5 (8.3)
Insurance issue involvement		
No	13 (21.7)	17 (28.3)
Not anymore	24 (40.0)	26 (43.3)
Yes	23 (38.3)	17 (28.3)
Litigation (lawsuit involvement)		
No	52 (86.7)	55 (91.7)
Yes	8 (13.3)	5 (8.3)
Compensation received for injury		
No	29 (48.3)	26 (43.3)
Yes	12 (20.0)	20 (33.3)
Pending	19 (31.7)	14 (23.3)
Previous whiplash injury		
No	44 (73.3)	38 (63.3)
Yes	16 (26.7)	22 (36.7)
Medication intake		
No	34 (56.7)	33 (55.0)
Yes	26 (43.3)	27 (45.0)

Abbreviations: cWAD, chronic whiplash-associated disorder; MPNA, modern pain neuroscience approach; UC, usual care.

^a^ Data were missing for 1 participant in the MPNA group.

^b^ Data were missing for 7 participants in the UC group and 1 in the MPNA group.

**Figure.**
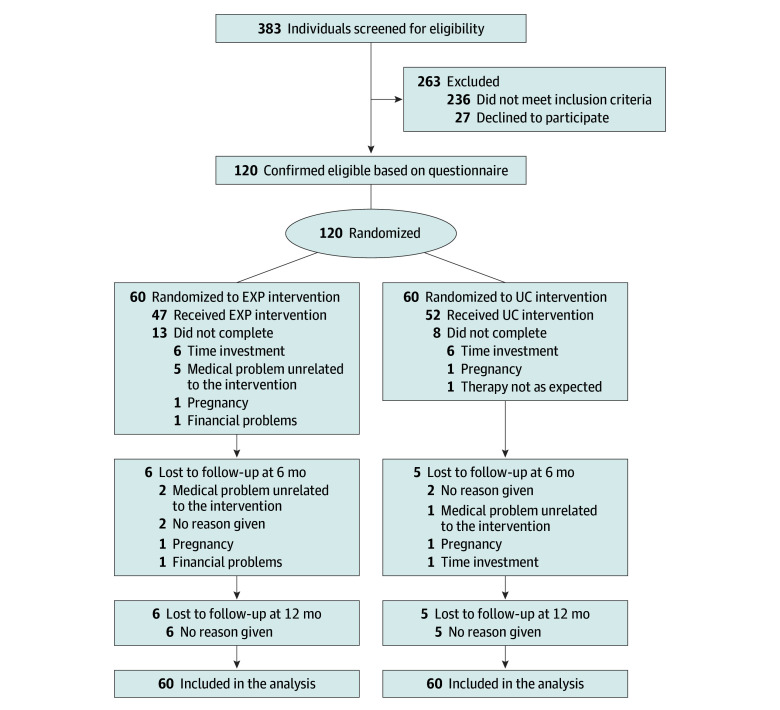
Study Flowchart A total of 120 individuals were included in the analysis because of the use of linear mixed-models analysis, with a likelihood-estimation procedure whereby likely values for missing data are estimated from information contained in the observed data, resulting in nonbiased estimates, provided that data are missing at random. Six-month follow-up was the primary end point. EXP indicates experimental treatment; UC, usual care.

Therapy adherence was checked, as each participant’s number of sessions was registered. The mean (SD) number of sessions, 16.2 (4.8) for UC and 15.8 (5.2) for MPNA, did not significantly differ between groups (mean group difference [MGD], −0.42 [95% CI, −2.30 to 1.46]; *P* = .48).

### Protocol Deviations

The protocol included electroencephalography (EEG) to assess brain activity during experimental pain assessments. EEG data were not included in the analysis because of substantial missing data due to (1) COVID-19 trial disruptions; (2) technical device issues; (3) participants’ dropout or discomfort with retaking the EEG protocol in follow-up; and (4) insufficient amount of usable data from deterioration in active signal quality, despite using various filtering methods and efforts to remove flat channels in the available EEG data.

### Effect of the Interventions

Table 3 shows the detailed results of all analyses. Analysis of the primary outcome, pain-related disability (NDI) at 6-month follow-up, showed no significant group differences, with an MGD of 2.38 points (95% CI, −0.12 to 4.89 points; *P* = .15; Cohen *d* = 0.646). In response to the intervention, mean neck pain–related disability decreased by 5.74 points (33.3%) in the MPNA group and 3.22 points (15.8%) in the UC group. However, a significant group difference was observed in favor of the MPNA group immediately after treatment (MGD, 3.55 [95% CI, 1.12-6.26]; *P* = .006; Cohen *d* = 0.999) and at the 12-month follow-up (MGD, 3.54 [95% CI, 0.81-6.28]; *P* = .02; Cohen *d* = 0.947). From a clinical point of view, the change in functional status from baseline to follow-up exceeded the MCID (ie, 3.5 of 50) in both groups. Notably, immediately after intervention and at the 12-month follow-up, the MGD exceeded the MCID in favor of the MPNA group, with values of 3.55 and 3.54. Furthermore, the MPNA group achieved a mean reduction in NDI score below the cutoff of 15.

**Table 3.** Clinical Effectiveness Outcomes, Adjusted for Age and Sex^a^

Time point	Participant group, mean (SE)		Mean group difference (95% CI)	Between-group comparison		Interaction effect^b^			Main effect of time	
	MPNA (n = 60)	UC (n = 60)		*P* value^c^	Cohen *d*	F value	*P* value^c^	Effect size, η^2^ value^d^	F value	*P* value^c^
**Primary outcome: functional status**										
Neck Disability Index score^e^										
T0	18.69 (0.67)	19.72 (0.69)	NA	NA	NA	2.63	.11	<0.001	38.77	.001
T1	12.95 (0.89)	16.50 (0.88)	3.55 (1.12 to 6.26)	.006	0.999					
T2	14.23 (0.93)	16.62 (0.91)	2.38 (−0.12 to 4.89)	.15	0.646					
T3	13.83 (1.02)	17.35 (0.98)	3.54 (0.81 to 6.28)	.02	0.947					
**Secondary self-reported outcomes**										
SF-36 Physical Status score, %^f^										
T0	31.97 (1.00)	32.69 (1.03)	NA	NA	NA	0.85	.36	<0.001	47.38	.001
T1	38.77 (1.12)	34.98 (1.11)	−3.79 (−5.00 to 1.47)	.04	0.687					
T2	36.28 (1.18)	36.23 (1.13)	−0.04 (−3.15 to 3.06)	>.99	0.009					
T3	38.37 (1.22)	36.60 (1.18)	−1.76 (−5.00 to 1.47)	.85	0.320					
SF-36 Mental Status score, %^f^										
T0	51.48 (1.76)	48.19 (1.82)	NA	NA	NA	<0.001	.95	<0.001	0.98	.32
T1	55.73 (1.94)	53.65 (1.92)	−2.08 (−7.25 to 3.08)	>.99	0.244					
T2	55.37 (2.02)	50.49 (1.96)	−4.88 (−10.20 to 0.44)	.22	0.571					
T3	52.16 (2.08)	50.20 (2.02)	−1.95 (−7.46 to 3.55)	>.99	0.229					
Numeric Pain Rating Scale score^b^										
T0	5.44 (0.25)	5.51 (0.26)	NA	NA	NA	0.11	.74	<0.001	27.13	.001
T1	3.28 (0.28)	3.95 (0.28)	.66 (−0.09 to 1.42)	.26	0.481					
T2	4.14 (0.29)	3.87 (0.29)	−0.27 (−1.05 to 0.51)	*>*.99	0.195					
T3	4.20 (0.30)	4.33 (0.30)	0.12 (−0.69 to 0.94)	*>*.99	0.090					
Patient Specific Complaints Questionnaire score^g^										
T0	20.21 (0.77)	19.82 (0.80)	NA	NA	NA	4.65	.03	0.02	16.11	.001
T1	14.97 (0.84)	15.90 (0.84)	0.93 (−1.33 to 3.19)	*>*.99	0.229					
T2	16.40 (0.88)	18.55 (0.87)	2.15 (−0.20 to 4.50)	.22	0.529					
T3	15.97 (0.91)	17.94 (0.90)	1.96 (−0.47 to 4.41)	.35	0.483					
Pain Catastrophizing Scale score^h^										
T0	24.24 (1.57)	25.43 (1.62)	NA	NA	NA	2.20	.14	<0.001	48.69	.001
T1	13.31 (1.70)	18.98 (1.71)	5.66 (1.10 to 10.22)	.045	0.764					
T2	15.53 (1.76)	20.06 (1.74)	4.52 (−0.15 to 9.21)	.17	0.610					
T3	13.75 (1.82)	18.57 (1.80)	4.82 (−0.03 to 9.68)	.16	0.650					
Pain Anxiety Symptoms Scale score^i^										
T0	31.43 (2.45)	35.49 (2.54)	NA	NA	NA	3.92	.048	0.01	15.27	.001
T1	18.37 (2.62)	29.64 (2.65)	11.26 (4.25 to 18.27)	.005	1.112					
T2	21.55 (2.70)	30.73 (2.69)	9.17 (2.00 to 16.34)	.04	0.906					
T3	21.44 (2.77)	32.49 (2.76)	11.05 (3.67 to 18.44)	.01	1.092					
Central Sensitization Inventory score^j^										
T0	44.69 (1.83)	48.85 (1.91)	NA	NA	NA	2.58	.001	0.04	22.53	.001
T1	37.08 (1.91)	43.88 (1.96)	6.79 (1.67 to 11.92)	.003	1.164					
T2	37.77 (1.95)	44.53 (1.98)	6.76 (1.56 to 11.96)	.03	1.158					
T3	36.15 (1.98)	46.86 (2.01)	10.71 (5.41 to 16.00)	.001	1.834					
Impact of Event Scale–Revised score^k^										
T0	16.13 (1.92)	19.86 (1.99)	NA	NA	NA	0.03	.85	<0.001	4.54	.03
T1	12.05 (2.08)	13.44 (2.09)	1.38 (−1.45 to 8.91)	*>*.99	0.157					
T2	13.83 (2.15)	16.31 (2.13)	2.47 (−4.17 to 6.94)	*>*.99	0.281					
T3	12.50 (2.22)	16.66 (2.19)	4.15 (−1.75 to 10.06)	.50	0.471					
**Secondary experimental pain-related outcomes**										
Electrical detection threshold on left wrist, mA										
T0	1.16 (0.09)	1.19 (0.09)	NA	NA	NA	0.78	.38	<0.001	8.39	.004
T1	1.21 (0.10	1.44 (0.10)	0.22 (−0.05 to 0.51)	.23	0.390					
T2	1.34 (0.11)	1.51 (0.11)	0.16 (−0.14 to 0.47)	.57	0.285					
Electrical detection threshold on right wrist, mA										
T0	1.18 (0.09)	1.21 (0.09)	NA	NA	NA	1.47	.23	<0.001	8.51	.004
T1	1.21 (0.10)	1.51 (0.10)	0.29 (0.01 to 0.58)	.08	0.519					
T2	1.33 (0.11)	1.54 (0.11)	0.20 (−0.10 to 0.51)	.37	0.358					
Electrical detection threshold on ankle, mA										
T0	1.76 (0.11)	1.81 (0.11)	NA	NA	NA	0.66	.42	<0.001	5.91	.02
T1	1.79 (0.13)	1.98 (0.12)	0.18 (−0.16 to 0.53)	.60	0.248					
T2	1.94 (0.14)	2.15 (0.13)	0.21 (−0.16 to 0.59)	.52	0.294					
Electrical pain threshold on left wrist, mA										
T0	5.04 (0.46)	5.39 (0.47)	NA	NA	NA	0.39	.53	<0.001	0.09	.75
T1	3.88 (0.46)	4.83 (0.47)	0.94 (−0.30 to 2.19)	.27	0.363					
T2	4.92 (0.54)	5.61 (0.53)	0.74 (−0.77 to 2.14)	.72	0.264					
Electrical pain threshold on right wrist, mA										
T0	5.37 (0.44)	5.65 (0.46)	NA	NA	NA	0.93	.34	<0.001	0.37	.55
T1	3.99 (0.44)	5.01 (0.46)	1.01 (−0.18 to 2.21)	.19	0.397					
T2	5.05 (0.53)	5.89 (0.52)	0.84 (−0.55 to 2.25)	.48	0.330					
Electrical pain threshold on ankle, mA										
T0	8.05 (0.61)	8.44 (0.63)	NA	NA	NA	0.14	.71	<0.001	0.86	.35
T1	5.88 (0.61)	7.01 (0.63)	1.12 (−0.53 to 2.79)	.38	0.323					
T2	7.81 (0.73)	8.35 (0.71)	0.53 (−1.40 to 2.48)	*>*.99	0.153					
Temporal summation on left arm										
T0	1.13 (0.25)	0.94 (0.25)	NA	NA	NA	2.76	.10	<0.001	0.08	.78
T1	0.67 (0.25)	0.90 (0.25)	0.23 (−0.43 to 0.91)	.98	0.155					
T2	0.89 (0.29)	1.38 (0.28)	0.48 (−0.28 to 1.25)	.43	0.319					
Temporal summation on right arm										
T0	0.91 (0.24)	1.15 (0.24)	NA	NA	NA	0.42	.52	<0.001	0.30	.58
T1	0.83 (0.24)	0.98 (0.25)	0.15 (−0.50 to 0.81)	*>*.99	0.102					
T2	0.90 (0.28)	1.44 (0.27)	0.53 (−0.21 to 1.29)	.32	0.352					
Temporal summation on ankle										
T0	1.35 (0.25)	1.72 (0.26)	NA	NA	NA	0.21	.83	<0.001	<0.001	.94
T1	1.08 (0.26)	1.45 (0.26)	0.37 (−0.32 to 1.07)	.59	0.226					
T2	1.49 (0.30)	1.62 (0.29)	0.13 (−0.67 to 0.93)	*>*.99	0.078					
Relative conditioned pain modulation on left arm, %										
T0	−7.69 (6.58)	−19.09 (6.81)	NA	NA	NA	0.05	.83	<0.001	0.13	.71
T1	−11.94 (7.72)	−20.63 (7.41)	−8.69 (−29.14 to 11.75)	.81	−0.186					
T2	−2.54 (8.32)	−17.72 (8.18)	−15.18 (−37.73 to 7.36)	.37	−0.326					
Relative conditioned pain modulation on right arm, %										
T0	−9.49 (5.40)	−11.36 (5.42)	NA	NA	NA	0.01	.94	<0.001	0.09	.76
T1	−13.71 (6.22)	−15.55 (6.12)	−1.83 (−18.37 to 14.70)	.83	−0.051					
T2	−6.64 (6.57)	−9.28 (6.72)	−2.64 (−20.73 to 15.45)	.77	−0.077					
Relative conditioned pain modulation on ankle, %										
T0	−8.96 (6.46)	−19.22 (6.47)	NA	NA	NA	0.29	.59	<0.001	0.81	.37
T1	−10.22 (7.25)	1.03 (7.18)	11.25 (−8.12 to 30.64)	.50	0.282					
T2	0.87 (7.69)	−19.75 (7.64)	−20.64 (−41.45 to 0.18)	.10	−0.516					

Abbreviations: MPNA, modern pain neuroscience approach; NA, not appliable; SF-36, 36-Item Short Form Health Survey; T0, baseline; T1, post intervention; T2, 6-month follow-up; T3, 12-month follow-up; UC, usual care.

^a^ Analyses were performed in R package ime4, version 1.1-35.4 (R Program for Statistical Computing), using an intention-to-treat linear mixed models approach assuming missing at random, with random intercept and restricted maximum likelihood. Treatment center was not maintained as random effect.

^b^ Scores range from 0 to 10, with higher scores indicating higher pain intensity.

^c^ Bonferroni corrected.

^d^ Effect sizes of interaction effects were computed to partial η^2^ as small (0.01), medium (0.06), and large (>0.14) using effect size package in R, version 1.1-35.4.

^e^ Scores range from 0 to 50, with scores of 15 or greater indicating disability.

^f^ Scores range from 0 to 100%, with higher scores indicating better functioning.

^g^ Scores range from 0 to 30, with higher scores indicating more inconvenience.

^h^ Scores range from 0 to 52, with higher scores indicating more catastrophizing.

^i^ Scores range from 0 to 100, with higher scores indicating higher pain anxiety symptoms.

^j^ Scores range from 0 to 100, with higher scores indicating more self-reported symptoms of central sensitization.

^k^ Scores range from 0 to 88, with higher scores indicating more distress.

On an explorative basis, similar analyses were conducted for the secondary outcome measures. The PASS revealed a significant group × time interaction effect (*P* = .048) with large effect sizes (Cohen *d* range, 0.906-1.112) and consistent, significant group differences at each time point favoring the MPNA group (MGD at immediate post treatment, 11.26 [95% CI, 4.25-18.27; *P* = .005; MGD at 6-month follow-up, 9.17 [95% CI, 2.00-16.34; *P* = .04]; MGD at 12-month follow-up, 11.05 [95% CI, 3.67-18.44; *P* = .01]). Similar results and large effect sizes (Cohen *d* range, 0.906-1.112) were observed for the Central Sensitization Inventory, with a significant interaction effect (*P* = .001) and consistent, significant group differences at each time point in favor of the MPNA group (MGD immediately after treatment, 6.79 [95% CI, 1.67-11.92; *P* = .003; Cohen *d* = 1.164]; MGD at 6-month follow-up, 6.76 [95% CI, 1.56-11.96; *P* = .03; Cohen *d* = 1.158]; MGD at 12-month follow-up, 10.71 [95% CI, 5.41-16.00; *P* = .001; Cohen *d* = 1.834]). Notably, only the MPNA group achieved a reduction in Central Sensitization Inventory scores below the cutoff of 40.

For completeness, it is worth noting the significant group differences immediately after treatment in favor of the MPNA group for SF-36 Physical Status (MGD, −3.79 [95% CI, −5.00 to 1.47]; *P* = .04; Cohen *d* = 0.687) and the Pain Catastrophizing Scale (MGD, 5.66 [95% CI, 1.10-10.22]; *P* = .045; Cohen *d* = 0.764). However, these differences did not persist at the 6- and 12-month follow-ups, with effect sizes considered medium.

All other secondary outcomes showed no significant group differences. However, for pain intensity, 14 (23.3%) individuals in the MPNA group were deemed free of pain (ie, scores of 0 or 1 of 10 on the 11-point numeric rating scale to assess pain intensity directly posttreatment) vs 3 (5.0%) in the UC group. No serious adverse events were reported. Five participants of the MPNA group dropped out due to a medical problem unrelated to the intervention.

### Sensitivity and Cost-Utility Analyses

Comprehensive results of the sensitivity analysis showed no notable findings (eTables 7 and 8 in Supplement 2). Data of 88 participants (42 in the MPNA and 46 in the UC groups) were available for the cost-utility analysis. After exclusion of 7 cases with extreme cost values (3 in the MPNA and 4 in the UC groups), the base-case analysis included 81 cases (39 in the MPNA and 42 in the UC groups) and resulted in a cost-saving for MPNA compared with UC (incremental cost, US −$854.01) and a small benefit in QALYs in favor of MPNA (incremental QALY, 0.035). The probabilistic sensitivity analysis resulted in 84% certainty for the incremental cost-effectiveness ratio to be situated in the southeast quadrant of the cost-effectiveness plane, that is, for MPNA to dominate UC. All details, including scenario analyses, are available in eFigures 1 to 4 and eTables 3 to 5 in Supplement 2. None of the scenario analyses yielded conclusions significantly different from those of the base-case analysis.

## Discussion

Our primary hypothesis in this RCT, that MPNA compared with UC would lead to greater improvements in pain-related disability at the 6-month follow-up in individuals with cWAD, was not supported. This primary hypothesis was based on findings of a previous trial,^18^ which used the same design and interventions but without the inclusion of stress management, in a broader population with chronic spinal pain (including 10% with cWAD)and demonstrated consistent and significant effects (on cognitions). While both trials similarly suggest that MPNA may outperform the comparator, the present study did not reach significance at the primary end point (ie, NDI at 6-month follow-up). This raises the possibility that extrapolating findings from a broader population with chronic spinal pain to the population with specific cWAD may not be appropriate and that cWAD could represent a more complex clinical presentation. Notably, participants with cWAD in the present study exhibited more severe baseline symptoms (ie, higher levels of catastrophizing and lower functional status) compared with those in the earlier trial. Whether these differences reflect inherent complexity in cWAD or are due to sampling variation remains unclear. Furthermore, an important methodological difference lies in the use of the NDI as the primary outcome in the present study, unlike the previous trial, which may also account for divergent findings due to differences in psychometric sensitivity.

However, in absolute values, the MPNA group exceeded the NDI MCID (3.5 points) at the 6-month follow-up, while the UC group did not. Moreover, significant and clinically relevant NDI group differences favoring the MPNA group were observed directly after the intervention and at the 12-month follow-up. These primary outcome findings align with those of Overmeer et al,^48^ who compared neck-specific exercises with and without a behavioral psychological approach in cWAD. They demonstrated a 28% reduction in pain-related disability in the behavioral group, sustained to 12 months (*P* < .01).^48^ The percentage reductions in our study are similar.

To explain the unexpected deviation in NDI results at the 6-month follow-up, evidence on relapse patterns and their timing can be important. Relapse is common during behavioral change, which is the core element of MPNA,^47^ as individuals learn to cope differently and increase (avoided) activities despite pain.^47^ Studies on the transtheoretical model suggest that relapse often occurs 3 to 6 months into the action phase, potentially aligning with our 6-month follow-up findings.^49,50,51^ However, this hypothesis is speculative and does not fully account for the significant group differences that re-emerge at the 12-month follow-up.

On an explorative basis, MPNA appeared to reduce PASS-20 scores consistently over time more than UC. The PASS-20 is a tool to assess fear and anxiety responses specific to pain.^46^ Consistent with our findings, Overmeer et al^48^ also reported a long-lasting reduction in anxiety in only their behavioral group. Pain-related fear and anxiety are well-established drivers of persistent pain and are hypothesized to play a central role in the success of behavioral interventions incorporating pain neuroscience.^18,52,53^ The significant reduction in PASS-20 scores observed only in the MPNA group suggests that MPNA may be effective in addressing cognitive processes associated with pain chronicity. Although our methodology does not permit definitive conclusions on this matter, we hypothesize that this effect may primarily be attributed to the integration of stress management strategies in this group, which were absent in UC.

The lack of a corresponding reduction in self-reported and experimental pain intensity, despite improvements in pain-related disability, fear, and anxiety, can be attributed to the functional focus of MPNA, which does not prioritize pain reduction as a treatment goal. This distinction is crucial, as it highlights that patients can experience less disability, fear, and anxiety without necessarily reporting lower pain levels. This message is positive and empowering for those dealing with chronic pain. A similar observation was made in a trial by Castro et al^54^ in chronic low back pain, where reduced disability after cognitive functional therapy did not correspond with a decrease in pain levels. Moreover, a meta-analysis that included members of our group^55^ confirms that reducing pain-related fear and catastrophizing can mediate treatment effects of psychologically based intervention on disability outcomes, without mediating effects on pain intensity.

In line with the effects on pain-related fear and anxiety, MPNA also appeared more effective than UC in reducing self-reported symptoms of central sensitization across all time points. The Central Sensitization Inventory, which was used for this outcome, was originally designed to indicate the presence of central sensitization.^56^ However, the absence of changes in pain measures in our results fuels the discussion about its applicability for detecting central sensitization in various populations.^57,58^ It seems plausible that the overlap between anxiety-related constructs in the Central Sensitization Index and the PASS explain the concurrent improvements in both.^57,58^

In sum, our results support the conclusion of Overmeer et al^48^: physiotherapist-led neck-specific exercises combined with a behavioral approach—herein represented by MPNA—can have a positive impact on disability and anxiety in people with cWAD. Moreover, our health economic analysis confirmed that MPNA, compared with UC, is cost-effective. Nevertheless, for future research it remains important to identify subgroups of people who might benefit most, as case studies show that some individuals respond exceptionally well and can even achieve full recovery.

### Strengths and Limitations

This study has several strengths. To our knowledge, it is the first triple-blind, multicenter, sufficiently powered RCT investigating the treatment effects of MPNA in cWAD, supported by a prepublished trial protocol^25^ and long-term follow-up. The trial used an active control intervention within balanced treatment arms, and minimal contextual treatment effects, thereby enhancing internal and external validity. Treatment fidelity was maintained through follow-up refresher sessions to prevent therapy drift.

However, some limitations should be considered. Current involvement in a compensation claim was not included as an exclusion criterion, although such involvement is known to have a negative impact on treatment outcomes, and the potential impact on the results cannot be overlooked.^59,60,61^ However, we specifically chose not to exclude these participants to reflect clinical conditions, thereby maximizing external validity. Another limitation of this study is the exclusion of non–Dutch-speaking participants. While this decision was made to enhance methodological rigor, given the importance of language in self-report measures and therapeutic communication, it does reduce the inclusivity of the sample and may limit generalizability to more linguistically diverse clinical populations.

Additionally, the data analysis included a substantial number of outcomes (n = 21), assessed across a maximum of 4 time points depending on the outcome. Multiple testing correction was not applied, a decision influenced by the sample size and the aim of minimizing type II errors. As a result, significant findings should be interpreted with caution, emphasizing patterns and effect sizes rather than mere statistical significance. Also, all questionnaires were administered in a fixed order, which did not allow us to control for potential test order effects (eg, fatigue or learning). Last, significant results for the secondary outcomes should be interpreted with caution, as the trial was not powered to detect these. Therefore, the observed findings are explorative.

## Conclusions

Findings of this RCT did not support the prespecified primary outcome at the primary end point, disability at the 6-month follow-up. Secondary analysis showed that MPNA was cost-effective and reduced disability immediately after treatment and at 12 months and better addressed fear avoidance and central sensitization symptoms. Overall, in the absence of qualitative evidence-based guidelines, MPNA shows promise for managing cWAD, but future research to optimize therapy and identify those who benefit most is warranted.

## Notes

Supplement 1.
Trial Protocol

Supplement 2. **eTable 1.** Eligibility Criteria **eMethods 1.** Quantitative Sensory Testing Protocol **eTable 2.** Training and Supervision of Therapists **eMethods 2.** Cost-Utility Analysis **eTable 3.** Unit prices for Medication Use During Follow-Up **eTable 4.** Unit Prices for Visits With Clinicians **eTable 5.** Descriptives of Costs and effects for the MPNA and UC Groups **eFigure 1.** Cost-Effectiveness Plane of the Probabilistic Base Case Cost-Utility Analysis **eFigure 2.** Cost-Effectiveness Plane for Probabilistic Scenario Analysis No. 1 **eFigure 3.** Cost-Effectiveness Plane for Probabilistic Scenario Analysis No. 2 **eFigure 4.** Cost-Effectiveness Plane for Probabilistic Scenario Analysis No. 3 **eTable 6.** Overview of Missing Data per Outcome and Time Point **eTable 7.** Sensitivity Analyses, Corrected for Baseline NDI Levels **eTable 8.** Sensitivity Analysis, Baseline Characteristics of Completers vs Dropouts
eReferences.

Supplement 3.
Data Sharing Statement
