# Supplementary material for: Pain Science Education, Stress Management, and Cognition-Targeted Exercise Therapy in Chronic Whiplash Disorders: A Randomized Clinical Trial
Source: JAMA Netw Open. 2025 Aug 12;8(8):e2526674. doi: 10.1001/jamanetworkopen.2025.26674 (PMC12344539; doi:10.1001/jamanetworkopen.2025.26674)
Supplement: Supplement 1. — Trial Protocol [file jamanetwopen-e2526674-s001.pdf]

Supplementary material: Study protocol.

Original title:
A contemporary neuroscience approach compared to biomedically focused education combined with symptom-contingent exercise therapy in people with chronic whiplash associated disorders: a randomized controlled trial protocol

Objectives:
The primary objective is to examine whether the contemporary neuroscience approach (incl. pain neuroscience education plus cognition-targeted exercise therapy and stress management) is more effective than conventional, best-evidence physical therapy (including biomedically focused neck school education plus symptom-contingent graded and active exercise sessions) in reducing disability (primary outcome measure) and improving quality of life, socio-economic factors, and illness perceptions, and decreasing pain, central sensitization, posttraumatic stress symptoms, pain catastrophizing, and pain-related fear (secondary outcome measures) in people with chronic WAD.

Design:
A multicentre, randomized, triple-blind, controlled two-arm (1:1) trial conducted at the University Hospital Brussels, University Hospital Ghent and a regional hospital (Sint-Jozelfkliniek Campus Bornem). Data will be collected at baseline, at post-intervention, and at 6 and 12 months follow-up.

Study population:
One-hundred-twenty patients with chronic whiplash associated disorders (cWAD) will be recruited. Eligibility criteria are outlined in the table 1.

Inclusion	Exclusion
Women and men aged between 18 and 65 years	Having suffered loss of consciousness for more than 1 minute after the whiplash trauma
Fluent Dutch Speaker	Suffering neuropathic pain with diagnosis of nerve injury
Having experienced a whiplash trauma (i.e. neck pain resulting from a motor vehicle crash or traumatic event) diagnosed by a doctor (grade II to III as defined by the Quebec Task Force scale) which causes pain for at least 3 months with a mean pain frequency of ≥ 3 days per week	History of neck or shoulder surgery in the past 3 years
	History of specific spinal surgery (e.g. surgery for spinal stenosis)
	Being pregnant now or within the past year
Moderate to severe pain-related disability, established by a score of ≥15/50 on the Neck Disability Index	Suffering from epilepsy, chronic widespread pain syndromes, a psychiatric, rheumatic, endocrinological, or cardiovascular disorder
Not starting new treatments or medication and continuing their usual care 6 weeks prior to and during study participation	History of neuroscientific based therapy in patient history, and concomitant therapies

Table 1. In- and exclusion criteria

Recruitment strategy:
To avoid selection bias, a broad recruitment strategy including different sources will be carried out. Participants will be recruited from the hospitals affiliated with the participating universities (UZ Brussels and UZ Ghent), a secondary care hospital (Sint-Jozefkliniek), and additional hospitals and universities. Recruitment methods will include social media, primary care practices, pharmacies, patient support group publications, radio, newspapers, magazines, symposia, health insurance companies, and district health centres. Other measures taken to avoid selection bias include: 1) the development of a centralized recruitment manual; 2) shared protocol among trained recruiters; 3) logging of the source of recruitment; 4) documentation of individuals screened, eligible and recruited; 5) stratification in randomization techniques; and 6) post hoc sensitivity analyses.

Sample size calculation:
Sample size calculation was performed with G*Power 3.1.9.2 focusing on the primary outcome measure (functional status) and primary endpoint (6 months follow-up) based on the therapy effects on functional status (difference in functional status between baseline and 6 months follow-up in a previous randomized controlled trial[1] with 2 balanced treatment arms and comparable control and intervention therapies in patients with chronic spinal pain (NCT03239938)), and accounting for a 25% loss to follow-up after 6 months (based on the loss to follow-up percentage of the finished trial).[1] Calculations were based on two-tailed testing, F-tests, ANOVA: Repeated Measures, within-between interaction, with alpha = 0.05 and a desired power of 0.80, with a partial eta squared = 0.032, effect size = 0.18, allocation ratio (N2/N1) = 1, number of groups = 2, number of measurements = 2 (taking into account baseline and primary endpoint), correlation = 0.2, and 25% loss-to-follow-up, resulting in 60 patients in the experimental group and 60 patients in the control group. The total sample size is 120 people with chronic WAD.

Randomisation:
Participants will be randomized in a 1:1 ratio between the intervention and control arms using a stratified permuted block allocation, with the treatment centre as the stratification factor and a block size of four. Randomization lists for the three treatment centres will be prepared by an independent investigator from the Biostatistics Unit who has no other involvement in the study. A single independent researcher, who is not involved in recruitment, assessments, or treatment, will perform the randomization of participants after the baseline assessment. This researcher will conceal the randomization from both patients and other researchers using opaque, sealed envelopes.

Outcome measures:
Primary outcome:
Functional status has been selected as the primary outcome measure, as individuals with chronic pain regard improved functionality as a crucial treatment goal. The Neck Disability Index (NDI), the most widely used self-report outcome measure for neck pain, will be employed to assess pain-related disability levels (0-50)[2]. The NDI is known for its sensitivity, validity, and reliability in evaluating self-reported disability[3,4].

Explorative, secondary outcomes:
- Self-reported health-related quality of life, measured using the SF-36;
- Self-reported pain, using a 0-10 numeric rating scale;
- Self-reported symptoms of central sensitization, using the Central Sensitization Inventory;
- Electrical detection and electrical pain thresholds with a constant current electrical stimulator (DS7A Digitimer);
- Endogenous pain facilitation assessed by a temporal summation paradigm;
- Endogenous pain inhibition assessed by a conditioned pain modulation paradigm;
- Quantitative Electroencephalography (QEEG) (Sienna digital EEG, EMS Biomedical, Korneuburg, Austria) will be recorded from 32 Sn surface electrodes using an electrode cap (Headcap, Expertise in Medical Solutions Biomedical, Korneuburg, Austria);
- Self-reported Pain Catastrophizing, using the Pain Catastrophizing Scale;
- Self-reported Illness Perceptions, using the Illness Perception Questionnaire – Revised;
- Self-reported post-traumatic stress, using the Impact of Event Scale – Revised;
- Self-reported pain-related fear and fear-avoidance behaviour, using the Pain Anxiety Symptoms Scale;
- Socio-economic factors, using self-reported questionnaire and data including health-care expenditure from publicly funded healthcare organizations.

Interventions:
Both groups receive 18 sessions of approx. 30 minutes over a period of 16 weeks. All sessions are delivered by physical therapists (Master of Science), and are one-on-one, individualized sessions (except for one group pain educational session of 1h) using principles of person-centered care and applying guidance towards self-management. The experimental intervention compromises 3 sessions of pain neuroscience education combined with 15 one-on-one cognition-targeted time-contingent exercise therapy sessions and stress management.[5] The control intervention compromises of 3 sessions of biomedically focused neck school education and 15 sessions of symptom-contingent

graded and active exercise therapy sessions. The primary distinction between the groups lies in the approach to treatment: the contemporary neuroscience group (biopsychosocial, time-contingent) will address cognitive aspects of pain, stress management, targeting the brain, and considering central sensitization, whereas the control group (biomedical, symptom-contingent) will not include these elements. Both interventions will involve the same number of sessions, equal contact time with the therapist, and home exercises. To minimize contamination between groups, therapists providing the experimental treatment will not be involved in the control intervention and vice versa. Interventions will take place at different times of the day. Therefore, patients will not have contact with each other in hospital waiting rooms.

Practical organization Usual Care Best-Evidence Physiotherapy (UC – control intervention)

Table 2 shows the detailed practical organization of the 16-week control intervention, consisting of three sessions of biomedically focused neck school education and 15 sessions of symptom-contingent graded and active exercise therapy sessions.

Week 1	Biomedically focused neck school education – 3 one-on-one sessions (one group session of 1 hour, an informational leaflet and home-based online session of 30 minutes, and 2 individual one-on-one sessions of 30 minutes)	
Week 2		
Week 3		
Week 4	Symptom-contingent graded exercise therapy (one-on-one, 30 minutes)	Symptom-contingent graded exercise therapy (one-on-one, 30 minutes)
Week 5	Symptom-contingent graded exercise therapy (one-on-one, 30 minutes)	Symptom-contingent graded exercise therapy (one-on-one, 30 minutes)
Week 6	Symptom-contingent graded exercise therapy (one-on-one, 30 minutes)	
Week 7	Symptom-contingent graded exercise therapy (one-on-one, 30 minutes)	
Week 8	Symptom-contingent graded exercise therapy (one-on-one, 30 minutes)	
Week 9	Symptom-contingent graded exercise therapy (one-on-one, 30 minutes)	
Week 10	Symptom-contingent graded exercise therapy (one-on-one, 30 minutes)	
Week 11	Symptom-contingent graded exercise therapy (one-on-one, 30 minutes)	
Week 12	Symptom-contingent graded exercise therapy (one-on-one, 30 minutes)	
Week 13	Symptom-contingent graded exercise therapy (one-on-one, 30 minutes)	
Week 14	Symptom-contingent graded exercise therapy (one-on-one, 30 minutes)	
Week 15	Symptom-contingent graded exercise therapy (one-on-one, 30 minutes)	
Week 16	Symptom-contingent graded exercise therapy (one-on-one, 30 minutes)	

Table 2. Organization of the therapeutic sessions in the control arm

Content

a. Biomedically focused neck school education

   I. *First session*

   The first session of biomedically focused neck school education was held as a group session of a maximum of 6 people, which lasted about 1 hour. Information was provided to the participants using a PowerPoint, and covered following topics: 1) What is a whiplash; 2) which complaints are prevalent after a whiplash; 3) The anatomy of the spine, including the location and function of the vertebra, discus, ligaments, joints, nerves, and muscles; 4) Movements of the neck; 5) Ergonomical principles applied to standing and sitting position; 6) An explanation of the chronic pain, using biomedical causes including damage of the spine, ligaments or disci, wear of the spine, muscle fatigue, posture of the spine, physical strain, problems in load capacity of the spine, and insufficient exercise and physical activity; and 7) how to tackle the chronic pain using education, ergonomics and a physical therapy program.

   The session was closed by giving practical instructions on the online module (see below) and by providing an information leaflet. This leaflet mirrored the precise content covered in the group session. The participants were directed to read the leaflet, make note of any questions that may arise, and ideally, share the leaflet with a significant other. This sharing aimed to ensure that participants could receive social support during the intervention. After closing the session, the therapist sends an e-mail to each of the participants including the link and instruction for the online module.

   II. *Online module*

   The online module consisted of a website-embedded slideshow. Each slide featured a single sentence accompanied by a relevant image. The overarching purpose of the complete slideshow was to give the same information as during the first session, but in

a slightly different format. Consequently, the content presented in the online slideshow mirrored the material covered in the group session.

III. *Second and third session – one-on-one*

The final two educational sessions in the control arm consisted of individual one-on-one sessions. These sessions covered the same information as the initial group session but were tailored to each participant. For instance, ergonomic principles were discussed with a focus on the participant's specific situation. Personal challenges in daily life, both at home and at work, were explored, and alternatives were suggested. Practical exercises were conducted to address specific scenarios, such as lifting, sitting, and transitioning from lying to standing. The implementation of these ergonomic principles in daily life was also reviewed. Additionally, the balance between load and load capacity was discussed, emphasizing situations and factors in the participant's life that increase spinal load. The last educational session also included an explanation of the symptom-contingent exercise approach and its rationale, defining a pain-free range of motion and its importance in therapy. Finally, goals for the exercise therapy sessions were discussed and established.

b. Symptom-contingent graded exercise therapy[6]

All 15 sessions of symptom-contingent graded exercise therapy lasted about 30 minutes and were organized as one-on-one sessions. Each session started with a warming-up, which included one circulatory exercise and two mobilizing exercises. The core of the exercise session first focused mainly on endurance and stability and later in the program switched towards muscle strength. Every session was closed by relaxation (first sessions) or stretching exercises (later sessions) as cooling down.

I. Examples of warming-up exercises:
   - Circulatory: Midrange exercises, 30 to 50 repetitions, focusing on cervical spine, scapulothoracic spine and shoulders. Exercises used included for example head sways, shoulder rolls, and shoulder shrugs.
   - Mobilizing: 3 times 20 seconds (grade to 3 times 45 seconds), rotation of the cervical spine, lateral flexion of the cervical spine, extension and flexion of the cervical spine,

II. Examples of endurance exercises:
3 times 25 repetitions per exercise, including exercises like ‘low row’, ‘elbow in the back pocket’, ‘serratus punch’, and ‘dynamic hug’.

III. Examples of stabilizing exercises:
100 repetitions spread over 3 bouts at 50% of 1RM, including exercises like ‘push-up at the wall’, ‘wall gliding’, ‘wall sliding’, ‘arm adduction’, and planking.

IV. Examples of muscle strength exercises:
3 times 8-12 repetitions, including exercises focusing on isometric neck muscle strength, neck extensors and flexors, stabilizing muscles, cervical rotation, etc.

Importantly, in this control arm, communication regarding the exercises and grading these exercises is done using a symptom-contingent approach. This entails that exercises are altered (e.g., lower intensity, frequency, number of repetitions, etc.) when the participant is reporting pain. From a communication point of view, the therapist will ask the participant to tell him/her when the exercise is perceived as too heavy or causing pain.

Practical organization Modern Pain Neuroscience Approach (MPNA – experimental intervention)

Table 3 shows the detailed practical organization of the 16-week experimental intervention, consisting of three sessions of pain neuroscience education and 15 sessions of cognition-targeted time-contingent exercise therapy sessions and stress management.

Week 1	Pain Neuroscience Education, including information on stress – 3 sessions (one group session of 1 hour, an informational leaflet and home-based online session of 30 minutes, and 2 individual one-on-one sessions of 30 minutes)	
Week 2		
Week 3		
Week 4	CTET and stress management (one-on-one, 30 minutes)	CTET and stress management (one-on-one, 30 minutes)
Week 5	CTET and stress management	CTET and stress management

	(one-on-one, 30 minutes)	(one-on-one, 30 minutes)
Week 6	CTET and stress management (one-on-one, 30 minutes)	
Week 7	CTET and stress management (one-on-one, 30 minutes)	
Week 8	CTET and stress management (one-on-one, 30 minutes)	
Week 9	CTET and stress management (one-on-one, 30 minutes)	
Week 10	CTET and stress management (one-on-one, 30 minutes)	
Week 11	CTET and stress management (one-on-one, 30 minutes)	
Week 12	CTET and stress management (one-on-one, 30 minutes)	
Week 13	CTET and stress management (one-on-one, 30 minutes)	
Week 14	CTET and stress management (one-on-one, 30 minutes)	
Week 15	CTET and stress management (one-on-one, 30 minutes)	
Week 16	CTET and stress management (one-on-one, 30 minutes)	
CTET = Cognition-Targeted Exercise Therapy		

Table 3. Organization of the therapeutic sessions in the experimental arm.

<u>*Content*</u>

a. Pain Neuroscience Education (PNE) (3 sessions)

i. *First session*

The first session of PNE was held as a group session of a maximum of 6 people, which lasted about 1 hour. PNE aims to reconceptualize the patients' pain beliefs, to increase the patients' knowledge of pain and to decrease its threat value. The content will be based on current knowledge of the neurophysiology of pain according to Wall and Melzack[7], 'Pijneducatie, een praktische handleiding voor de (para)medici' by Van Wilgen and Nijs[8], and 'Explain Pain' by Butler and Moseley[9], including following topics in laymen's terms: (1) the neuron, (2) the synapse, (3) descending nociceptive inhibition and facilitation, (4) peripheral sensitization, (5) central sensitization, as well as (6) an explanation about stress, stress mechanisms/responses, and the relationship between stress and pain.

Information was provided to the participants using a PowerPoint, during which specific questions were asked to the participant (e.g., "Wat symptoms do you have, and how do you cope with them right now?", "What comes to mind when hearing the terms acute and chronic pain, does that ring a bell?", "Can you explain in your own words what the nervous system is?"). These questions had several aims: they ensure active participation of the participants, lower the barriers to ask questions, and allow the therapist to individualize the content to the group. By the end of the session the goal was that participants were convinced that pain is not the direct result of tissue damage, but rather linked to hypersensitivity of the peripheral and central nervous system.

The session was closed by giving practical instructions on the online module and by providing an information leaflet. This leaflet mirrored the precise content covered in the group session. The participants were directed to read the leaflet, make note of any questions that may arise, and ideally, share the leaflet with a significant other. This sharing aimed to ensure that participants could receive social support during the intervention.

In this session, the completed feared activity forms were collected. Participants had initially received these forms during the baseline assessment, with instructions to complete them before the first therapy session. This timing was intentional to ensure that the information provided during this initial session did not influence the completion of the documents. Further details regarding the content and utilization of the feared activities form can be found in the description of the third educational session.

After closing the session, the therapist sends an e-mail to each of the participants including the link and instruction for the online module.

ii. *Online module*

The online module consisted of a website-embedded slideshow. Each slide featured a single sentence accompanied by a relevant image. The overarching purpose of the complete slideshow was to challenge misconceptions and erroneous beliefs associated with pain. Consequently, the content presented in the online slideshow mirrored the material covered in the group session.

Following the completion of the slideshow, the participants were asked to fill out a questionnaire. It's important to note that this questionnaire did not serve as an outcome measure in the trial; rather, it functioned exclusively as a practical tool for the therapist to tailor the content of the third educational session. The first part of this questionnaire featured the Neurophysiology of Pain test, designed to assess participants' understanding of the subject matter. This aspect helps identify specific areas that may require more in-depth discussion to ensure a comprehensive grasp of PNE. The second part of the questionnaire comprised open-ended questions to assess the participant's beliefs regarding their symptoms. Some examples are: "Do you believe that tissue damage is the root cause of your chronic pain issue?" "Do you think your nervous system is hypersensitive, and if so, what factors contribute to that?" "What actions can you take to exacerbate/alleviate symptoms?" and "Can your social environment play a role, and how does it cope with your chronic pain?".

iii. Second and third session – one-on-one

The third educational session was organized as an individual face-to-face session between the therapist and the participant. To assist therapists, a session template was provided to guide the discussion in these sessions, and ensured focus on illness perceptions of the participants, the aim and rationale of stress management, three different strategies to deal with stress, the aim and rationale of cognition-targeted exercise therapy, the aim and rationale for a time-contingent approach, goal setting for exercise therapy based on the feared activities form. Goals were generally formulated SMART, and were always functional, never focusing on pain reduction or alleviation as pain is not a reliable symptom. If the patient wanted to focus on pain in the goals, the therapist asked questions on the content of the PNE sessions to allow the participant to understand that focusing on pain does not match the newly derived knowledge. It is essential to emphasize that not all aspects outlined in the template were obligatory for discussion, and the therapist had the flexibility to personalize the session by responding to the unique needs and narrative of the participant.

b. Cognition-targeted exercise therapy and stress management (15 sessions)

i. Cognition targeted exercise therapy (CTET)

All exercise therapy sessions were individual, one-one-one sessions between the participant and the therapist, each lasting about 30 minutes. The therapists were free to choose specific exercises, if they fitted the gradual progression towards the functional goals and if they followed cognition-targeted principles[10]. These principles included[5]:

- All exercises are performed in a time-contingent manner ("Perform this exercise 10 times, regardless of the pain") rather than in a symptom-contingent way ("Stop or adjust the exercise when it hurts").
- Valued goal setting is done together with the patient, and the goals are focused on functionality instead of pain relief.
- The treating therapist continuously questions and challenges the patients' pain cognitions and perceptions and the expected outcome of each exercise, to change unhelpful cognitions and perceptions into positive ones. Instructions on the applied communication techniques are described extensively elsewhere[11].
- Exercises progress towards more feared movements and activities, and fears and unhelpful perceptions on negative consequences are discussed in this regard.
- Progression to more feared exercises is preceded by a phase of motor imagery, in patients with a high fear-level.

The purpose of CTET is to confront the patient with movements and activities that are feared, avoided and/or painful[5]. It was essential, both in physiotherapy sessions, during home exercises, and in daily life, to steer clear of all 'safety behaviours' and emphasize normal and functional movements in a relaxed manner (i.e., without consciously contracting specific muscles or employing maladaptive compensation strategies). The progression aimed at advancing to more complex and more feared movements and activities. Naturally, this progression was tailored to each individual patient based on their specific feared movements. Typically, the initial exercises mirrored the movement intended for the final activity but without triggering an association with the actual feared or

painful movement. Ideally, these initial exercises induced some level of fear or stress to promote a successful learning experience leading to increased confidence.

Again, it should be acknowledged that communication on cognitions, perceptions and expectations was an essential component of cognition-targeted exercises as described above. The exercises were not used to target local aspects of the neck/back but were rather behavioural experiments targeting the brain. Specific examples of cognition-targeted communication during exercises between the physiotherapist and a chronic pain patient are extensively described elsewhere[11].

Several exercises, movements and activities used in the exercise program were also practiced at home. Therapists were instructed to use shared-decision principles to decide upon number, intensity and frequency of exercises, considering what exercises had been performed under the supervision of the therapist and what the participant considered feasible. It is crucial for the home exercises to be sufficiently challenging, yet the patient should feel confident to perform them independently at home. All home exercises were assigned with a focus on functionality rather than pure analytical movements and were applied using a time-contingent approach5. Moreover, communication on home exercises was equally important as communication during the exercises in therapy. At the start of each cognition-targeted exercise session, the last session and the home exercises were discussed, allowing to check therapy adherence, but also to check for potential problems or deterioration into old ‘biomechanical’ beliefs considering the pain problem. Upon occurrence, unhelpful beliefs were tackled by referring to the PNE sessions and by questioning the doubts of the participant.

During the very last session, the participants were provided with a document including the general principles of a cognition-targeted time-contingent exercise approach, as well as some examples of exercises that were performed during the intervention to ensure that they were able to maintain active after the end of therapy.

c. Stress management

Stress management exercises and techniques were continuously integrated in the PNE and CTET program. Table 4 illustrates the specific organization.

The first step involves the education phase. During the individual education session, the therapist will inquire about the patient's experience with stress and the extent of it, as well as their current stress coping strategies. The therapist will then introduce various stress management techniques, including Jacobson's progressive relaxation therapy, visualization, and mindfulness. The participant will select one of these techniques to practice at home in a quiet environment. Ultimately, the patient will choose one technique to follow for the ongoing stress management protocol.

The next phase, known as the initiation phase, involves several key steps. First, the participant selects a stress management technique to try out at home and identifies relevant stressors in their life. Following this, the therapist engages the participant in a discussion about their perceptions of stress and introduces various coping strategies. Together, they determine which coping strategies will be most effective for each identified stressor.

Afterwards, the participant receives a stress reaction record to complete at home. Subsequently, during the skills training phase, the participant practices their chosen stress management technique during quiet moments. As they progress, the participant begins to apply the technique in increasingly challenging and stressful situations, which marks the confrontation phase.

Week 1	Therapy session 1 (PNE)	**Education phase of stress management:**<br>• Explanation about stress, stress mechanisms/responses, relationship between stress and pain<br>• The therapist explains the 3 different stress management techniques and the patient chooses one technique to try at home.<br>• The aim of stress management (i.e. to learn how to cope better
Week 2	Therapy session 2 (PNE)	
Week 3	Therapy session 3 (PNE)	

		with daily stressors and to reduce the negative consequences of stress) will be explained.<br>**Initiation phase of stress management:**<br>• List relevant stressors of the patient (identification of stressors)<br>• The therapist checks the patients' perceptions about stress<br>• The therapist identifies the current stress coping strategies of the patient and explains 3 different stress coping strategies (avoiding, changing, and learning to handle the stressor(s)).
Week 4	Therapy session 4 (CTET)	**Initiation phase of stress management (continued):**<br>• The patient tries different techniques (Jacobson, mindfulness, visualization) → choice of technique<br>• The therapist explains the use of the stress reaction record and gives the record to the patient to fill out at home to obtain insight in the thoughts, emotions, and coping strategies in response to stressors.<br>• The therapist evaluates the stress reaction record.
	Therapy session 5 (CTET)	
Week 5	Therapy session 6 (CTET)	
	Therapy session 7 (CTET)	
Week 6	Therapy session 8 (CTET)	
Week 7	Therapy session 9 (CTET)	**Skill training phase of stress management:**<br>• The participant practices stress management technique during quiet non-stressful moments<br>• The therapist evaluates the stress reaction record.
Week 8	Therapy session 10 (CTET)	
Week 9	Therapy session 11 (CTET)	
Week 10	Therapy session 12 (CTET)	
Week 11	Therapy session 13 (CTET)	
Week 12	Therapy session 14 (CTET)	**Confrontation phase of stress management:**<br>• The participant performs the stress management technique during increasingly challenging and stressful situations (gradual exposure)<br>• The therapist evaluates the stress reaction record.
Week 13	Therapy session 15 (CTET)	
Week 14	Therapy session 16 (CTET)	
Week 15	Therapy session 17 (CTET)	
Week 16	Therapy session 18 (CTET)	**End of guided stress management:** The therapist guides the participant regarding further application of stress management in daily life without supervision.

Table 4. Illustration of the integration of stress management within the CTET sessions.

Statistical analysis plan

1. **Data source:** Data are derived from participant suffering from cWAD who participate in this randomised controlled trial. They were evaluated at baseline, at post-intervention and at 6 and 12 months follow-up. The primary outcome is functional status (disability), assessed using the Neck Disability Index.
2. **Analysis objectives:** To investigate the effectiveness of a contemporary neuroscience approach (incl. pain neuroscience education plus cognition-targeted exercise therapy and stress management) versus conventional, best-evidence physical therapy (including biomedically focused neck school education plus symptom-contingent graded and active exercise sessions) in terms of the above-mentioned primary outcome at 6 months follow-up, and quality of life, socio-economic factors, and illness perceptions, and decreasing pain, central sensitization, posttraumatic stress symptoms, pain catastrophizing, and pain-related fear (secondary outcome measures).
3. **Population:** People suffering from cWAD
4. **Endpoints:** Change in outcome measures from baseline to each follow-up point
5. **Covariates:** Models will be adjusted for age and sex.

6. **Handling of missing data:** Analyses are carried out using linear mixed models which can handle missing data. Intention to treat analysis is generally favoured because it avoids bias associated with non-random loss of participants and corresponds to analysing the groups exactly as randomized regardless of whether they received the randomized treatment. To avoid selection bias, we will perform an 'as randomized' analysis which retains participants in the group to which they were originally allocated. To prevent attrition bias, outcome data obtained from all participants will be included in the data analysis. These two conditions (i.e., all participants, as randomized) define our intention to treat analysis, which is recommended as the preferred analysis strategy.
7. **Statistical methodology:**
   Descriptive statistics will be used to report the demographic variables and baseline characteristics of the participating cWAD patients per intervention group, with median and interquartile range. All analyses were by intention-to-treat, with all participants included in the analysis according to their randomly allocated treatment. All analyses will be performed in R. Linear mixed models with random intercept and restricted maximum likelihood will be fitted for each outcome. The models will include fixed effects for timepoint, treatment allocation, and their interaction; adjusted for age and sex. Effect sizes will be computed as partial eta-squared (η^2). Estimated marginal means will be calculated per timepoint and treatment arm. $P < .05$ (two-sided) will be deemed as significant. Additionally, the percentage of patients per group that exceed the MCIC for the primary outcome measure will be calculated. We will consider a between-group difference of 3.5 points on disability (NDI) and 1 point on pain intensity (NPRS) to be minimal clinically important.

References

1. Malfliet A, Kregel J, Coppieters I, et al. Effect of Pain Neuroscience Education Combined With Cognition-Targeted Motor Control Training on Chronic Spinal Pain: A Randomized Clinical Trial. *JAMA Neurol*. Jul 1 2018;75(7):808-817. doi:10.1001/jamaneurol.2018.0492
2. Vernon H. The Neck Disability Index: state-of-the-art, 1991-2008. *J Manipulative Physiol Ther*. Sep 2008;31(7):491-502. doi:10.1016/j.jmpt.2008.08.006
3. Jorritsma W, de Vries GE, Dijkstra PU, Geertzen JH, Reneman MF. Neck Pain and Disability Scale and Neck Disability Index: validity of Dutch language versions. *Eur Spine J*. Jan 2012;21(1):93-100. doi:10.1007/s00586-011-1920-5
4. Jorritsma W, de Vries GE, Geertzen JH, Dijkstra PU, Reneman MF. Neck Pain and Disability Scale and the Neck Disability Index: reproducibility of the Dutch Language Versions. *Eur Spine J*. Oct 2010;19(10):1695-701. doi:10.1007/s00586-010-1406-x
5. Malfliet A, Kregel J, Meeus M, et al. Applying contemporary neuroscience in exercise interventions for chronic spinal pain: treatment protocol. *Brazilian Journal of Physical Therapy*. 2017;21doi:10.1016/j.bjpt.2017.06.019
6. ROBSON S, GIFFORD L. *Manual Therapy in the 21 st Centuty*. 2006.
7. Wall P, Melzack R. Textbook of Pain. 1999;
8. van Wilgen CP, Nijs J. Pijneducatie - een praktische handleiding voor (para)medici. 2010;
9. Butler DS, Moseley GL. Explain Pain. 2003;
10. Nijs J, Meeus M, Cagnie B, et al. A modern neuroscience approach to chronic spinal pain: combining pain neuroscience education with cognition-targeted motor control training. *Physical therapy*. 2014;94:730-738. doi:10.2522/ptj.20130258
11. Nijs JJ, Lluch Girbes E, Lundberg M, et al. Exercise therapy for chronic musculoskeletal pain: Innovation by altering pain memories. *Manual therapy*. 2015;20:216-220. doi:10.1016/j.math.2014.07.004
