# Supplementary material for: Pain Science Education, Stress Management, and Cognition-Targeted Exercise Therapy in Chronic Whiplash Disorders: A Randomized Clinical Trial
Source: JAMA Netw Open. 2025 Aug 12;8(8):e2526674. doi: 10.1001/jamanetworkopen.2025.26674 (PMC12344539; doi:10.1001/jamanetworkopen.2025.26674)
Supplement: Supplement 2. — eTable 1. Eligibility Criteria eMethods 1. Quantitative Sensory Testing Protocol eTable 2. Training and Supervision of Therapists eMethods 2. Cost-Utility Analysis eTable 3. Unit prices for Medication Use During Follow-Up eTable 4. Unit Prices for Visits With Clinicians eTable 5. Descriptives of Costs and effects for the MPNA and UC Groups eFigure 1. Cost-Effectiveness Plane of the Probabilistic Base Case Cost-Utility Analysis eFigure 2. Cost-Effectiveness Plane for Probabilistic Scenario Analysis No. 1 eFigure 3. Cost-Effectiveness Plane for Probabilistic Scenario Analysis No. 2 eFigure 4. Cost-Effectiveness Plane for Probabilistic Scenario Analysis No. 3 eTable 6. Overview of Missing Data per Outcome and Time Point eTable 7. Sensitivity Analyses, Corrected for Baseline NDI Levels eTable 8. Sensitivity Analysis, Baseline Characteristics of Completers vs Dropouts eReferences. [file jamanetwopen-e2526674-s002.pdf]

Supplementary Online Content

Malfliet A, Lenoir D, Murillo C, et al. Pain science education, stress management, and cognition-targeted exercise therapy in chronic whiplash disorders: a randomized clinical trial. *JAMA Netw Open.* 2025;8(8):e2526674. doi:10.1001/jamanetworkopen.2025.26674

eTable 1. Eligibility Criteria
eMethods 1. Quantitative Sensory Testing Protocol
eTable 2. Training and Supervision of Therapists
eMethods 2. Cost-Utility Analysis
eTable 3. Unit prices for Medication Use During Follow-Up
eTable 4. Unit Prices for Visits With Clinicians
eTable 5. Descriptives of Costs and effects for the MPNA and UC Groups
eFigure 1. Cost-Effectiveness Plane of the Probabilistic Base Case Cost-Utility Analysis
eFigure 2. Cost-Effectiveness Plane for Probabilistic Scenario Analysis No. 1
eFigure 3. Cost-Effectiveness Plane for Probabilistic Scenario Analysis No. 2
eFigure 4. Cost-Effectiveness Plane for Probabilistic Scenario Analysis No. 3
eTable 6. Overview of Missing Data per Outcome and Time Point
eTable 7. Sensitivity Analyses, Corrected for Baseline NDI Levels
eTable 8. Sensitivity Analysis, Baseline Characteristics of Completers vs Dropouts
eReferences.

This supplementary material has been provided by the authors to give readers additional information about their work.

© 2025 Malfliet A et al. *JAMA Network Open*.

eTable 1. Eligibility Criteria

Inclusion criteria	Exclusion criteria
a. Women and men aged between 18 and 65 years<br>b. Native Dutch Speaker<br>c. Having experienced a whiplash trauma (i.e. neck pain resulting from a motor vehicle crash or traumatic event) diagnosed by a doctor (grade II to III as defined by the Quebec Task Force scale) which causes pain for at least 3 months with a mean pain frequency of ≥ 3 days per week<br>d. Moderate to severe pain-related disability, established by a score of ≥15/50 on the Neck Disability Index<br>e. Not starting new treatments or medication and continuing their usual care 6 weeks prior to and during study participation	a. Having suffered loss of consciousness for more than 1 minute after the whiplash trauma<br>b. Suffering neuropathic pain with diagnosis of nerve injury<br>c. History of neck or shoulder surgery in the past 3 years<br>d. History of specific spinal surgery (e.g. surgery for spinal stenosis)<br>e. Suffering from epilepsy, chronic widespread pain syndromes, a psychiatric, rheumatic, endocrinological, or cardiovascular disorder<br>f. Being pregnant now or within the past year<br>g. History of neuroscientific based therapy in patient history, and concomitant therapies

© 2025 Malfliet A et al. *JAMA Network Open*.

eMethods 1. Quantitative Sensory Testing Protocol

Endogenous pain modulation was assessed with a quantitative sensory testing protocol including the determination of electrical detection thresholds and electrical pain thresholds, as well as the evaluation of endogenous pain modulation (focussing on both facilitatory and inhibitory pathways)[1-4].

a. Electrical detection threshold (EDT) and electrical pain threshold (EPT)
A Digitimer DSA7 constant current electrical stimulator was used to deliver electrical stimuli to the median nerve of both arms, and unilaterally to the sural nerve (ipsilateral to the painful neck side in case of unilateral neck pain, or to the dominant hand side in case of bilateral neck pain). For stimulation of the sural nerve, the felt pad electrode was placed 2 cm posterior to the lateral malleolus, whereas the cathode of this electrode was placed 5 cm proximally from the wrist (and the anodal electrode was placed 3 cm distally from the cathode) for stimulation of the median nerve[5]. Each stimulus consisted of a constant current rectangular pulse train of 5 pulses[6] delivered at a frequency of 250 Hz, each lasting 0.5 msec (with an inter stimulus interval of 3.5 msec and a total duration of the 5 pulse train of 20 msec)[7,8].
The order of the test locations (right wrist, left wrist, and ankle) was randomized. Stimuli started at an intensity of 0 mA and were gradually increased using steps of 0.5 mA[9,10] until the patient reported a faint sensation (i.e., EDT). Three consecutive measurements were taken at each site, with a 30 seconds interval to compute an average. The same procedure was then applied to determine the EPT, but with the instruction to report when the experience became unpleasant[5,11]. EPTs have been shown to be reliable to evaluate the sensitivity of the spinal nociceptive pathways in people with chronic pain[12].

b. Endogenous pain facilitation
Temporal summation (TS) of electrical pain was used as a measure of endogenous pain facilitation[13], during which 20 consecutive electrical stimuli[9] were delivered at the (previously determined) intensity of the EPT[11]. Stimuli were delivered with an inter-stimulus interval of 0.5sec and participants were asked to rate the 1st, 10th and 20th stimulus on a verbal numerical pain rating scale (NPRS) ranging from 0 (no pain) to 10 (worst imaginable pain)[9].

c. Endogenous pain inhibition
The efficacy of endogenous pain inhibition was assessed using a conditioned pain modulation (CPM) paradigm[14,15]. CPM is a reliable measure with intersession reliability varying from fair to excellent[16].

© 2025 Malfliet A et al. *JAMA Network Open*.

A cold pressor task, existing of immersion of the hand in water of 12°C during 3 minutes(64,70), functioned as the conditioning stimulus, whereas the application of 20 electrical stimuli (250 Hz, train of five, with a variable interstimulus interval of 8–12 seconds) at an intensity of 1.4 times EPT[17] formed the test stimulus[18]. Electrical stimuli were delivered unilaterally at the sural nerve and bilaterally at the median nerve[11], and were administered once before (baseline condition) and once during the application of the conditioning stimulus. Participants were asked to score the overall experience of the 20 electrical stimuli on a verbal NPRS ranging from 0 (no pain) to 10 (worst imaginable pain). If participants were not able to endure the cold water immersion and retracted their hand, the trial was registered as missing data.

© 2025 Malfliet A et al. *JAMA Network Open*.

eTable 2. Training and Supervision of Therapists

All therapy sessions were provided by experienced master-level physiotherapists specifically trained in the study interventions. To avoid treatment contamination, therapists are only trained in the intervention for their group and cannot switch groups after training. Usual Care (UC) therapists are not allowed to have prior training in the Modern Pain Neuroscience Approach (MPNA). Participants were always treated by the same trial therapist during the complete intervention. All training was based on standardized intervention manuals and followed a standardized structure.

**MPNA Therapists**	**UC therapists**
General face-to-face session (1,5h) containing:<br>1. Information on the study organization<br>2. Flow of the study participants<br>3. Timeline of the study and therapy<br>4. Principles of good clinical practice<br>5. Treatment manuals<br>6. Content and structure of the sessions<br>7. Demonstration of Pain Neuroscience Education (PNE) presentation<br><br>Training on MPNA included:<br>1. Individual preparation at home to perform PNE in a mock situation together with the trainers (approx. 2h).<br>2. Individual face-to-face session (3h) including mock PNE presentation with ad hoc feedback from the trainers. This session also covered the principles and content of cognition-targeted exercise therapy, with some case examples.<br>3. Individual preparation at home to master principles and content of CTET (approx. 2h).<br>4. Individual face-to-face session (2h) to cover CTET using case examples and an extensive Q&A.	General face-to-face session (1,5h) containing:<br>1. Information on the study organization<br>2. Flow of the study participants<br>3. Timeline of the study and therapy<br>4. Principles of good clinical practice<br>5. Treatment manuals<br>6. Content and structure of the sessions<br>7. Demonstration of biomedically focused neck school education presentation<br><br>Training on UC included:<br>1. Individual preparation at home to perform the biomedically focused neck school education in a mock situation together with the trainers (approx. 2h).<br>2. Individual face-to-face session (3h) including mock biomedically focused neck school education presentation with ad hoc feedback from the trainers. This session also covered the principles and content of symptom-contingent exercise therapy, with some case examples.<br>3. Individual preparation at home to master principles and content of symptom-contingent exercise therapy (approx. 2h).

© 2025 Malfliet A et al. *JAMA Network Open*.

	4. Individual face-to-face session (2h) to cover symptom-contingent exercise therapy using case examples and an extensive Q&A.

Every 8 months, a dedicated session was arranged for therapists (EXP and CON) and trainers to review and address any issues, to avoid any therapy drift, as well as to offer additional information or training as required. Moreover, in case of problems, doubts or any questions, the therapists were in close contact with the trainers through e-mail. If needed, additional online meetings were planned to tackle any issue.

Additionally, the therapists were instructed to use checklists specifically constructed for each therapy session to ensure that no aspects were forgotten. Moreover, the specific content of each therapy session related to MPNA or UC was captured by the therapists in a written lab notebook.

© 2025 Malfliet A et al. *JAMA Network Open*.

eMethods 2. Cost-Utility Analysis

1.1. Detailed method of Cost-Utility Analysis

The cost-utility analysis (CUA) was conducted using the Belgian Health Care Knowledge Centre (KCE) guidelines[19], EUnetHTA guidelines[20] and the handbook developed by Drummond et al. (4th edition)[21], and reported in line with the Consolidated Health Economic Evaluation Reporting Standards (CHEERS)[22]. CUA assessments took place at baseline, immediately post-intervention and at 6 and 12 months follow-up, resulting in a time horizon of 12 months post-intervention.

<u>Outcome measures for CUA</u>

Resource utilization and costs

The base case CUA was performed from a societal perspective, taking the following costs into account: (1) Direct costs related to healthcare visits during the follow-up period; (2) Direct costs related to medication use; and (3) productivity loss costs. The cost of intervention was not included in the CUA as this was equal for both groups (i.e., cost of 18 standard physiotherapy visits as determined by the Belgian National Institute for Health and Disability Insurance (NIHDI; unit price: USD 29.6), being USD 532), resulting in an incremental intervention cost of zero. Healthcare use (HCU) was not assessed during the intervention period; hence the reported healthcare costs only represent HCU during the post-intervention period. All costs were initially determined in Euros 2022 and converted to USD for reporting using the conversion rate of January 1, 2022 (coversion rate: $1 USD = €0.8792). If necessary, prices were adjusted using the health index for Belgium[23] in accordance with the guideline for economic evaluations in healthcare of the KCE[19].

HCU (i.e., medication use and visits with healthcare providers) was recorded using a retrospective self-reported questionnaire with 6 months recall at 6 and 12 months post-intervention[24,25]. The impact of the intervention on costs was expected to be related primarily to medication use and visits with healthcare providers, therefore costs related to hospitalizations were omitted from the CUA. Regarding medication use, participants registered: brand or substance name; dosage; and number of units taken. Costs related to medication use were subsequently determined by multiplying the unit cost by the number of units taken[19]. Unit costs for each type of medication were calculated by dividing the 2022 price of the least expensive package (according to the Belgian Centre for Pharmacotherapeutic Information (BCFI)[26]) by the number of units. A list of unit costs for medication can be found in eTable 3. Furthermore, participants reported their visits with healthcare providers (type of provider and number of visits). Healthcare visits were valued using unit prices for the corresponding NIHDI nomenclature codes (see eTable 4)[19,27].

© 2025 Malfliet A et al. *JAMA Network Open*.

At baseline, eligibility for work resumption and job type (if applicable) were assessed. Productivity loss during follow-up was evaluated using a retrospective questionnaire at 6 and 12 months follow-up that assessed the total duration of productivity loss in days. Productivity loss costs were valued by multiplying the number of days off-work by 7.6 (average work day length in hours) times the average labor cost of USD 49.5/hour for Belgium (i.e., Human Capital Approach[19]). For participants that were not eligible for work resumption, productivity loss costs were set to zero.

Utilities and QALYs

Participants completed a generic health-related quality of life measure, the Dutch version of the SF-36[21,28,29], that contains questions on 8 different health domains (physical functioning, role limitations due to physical or emotional problems, bodily pain, vitality, social functioning, and mental health) at baseline, immediately post-intervention, and at 6 and 12 months post-intervention. Based on these data SF-6D utility scores were derived using the algorithm developed by Brazier and Roberts (2004) which applies standard gamble health state valuation models[30]. SF-6D utility scores can vary between 0.29 and 1.00, with the latter indicating full health[31].
The QALY was determined for each participant by calculating the area under the curve when plotting the utilities over time[21].

Data processing and analyzes

All data processing and statistical analyses were performed in Microsoft Excel version 16.89.1 (Microsoft Corporation, Redmont, WA, USA) and SPSS version 29 (SPSS Inc., Chicago, IL, USA). Missing healthcare cost data was imputed with the median value for the respective comparator group for the respective timepoint.

Incremental cost-effectiveness ratio

Incremental cost-effectiveness ratios (ICER) for the base case and scenario analyses were calculated by dividing the incremental costs (mean costs for MPNA-experimental intervention minus mean costs for UC-control intervention) by the incremental effects (mean QALY for MPNA minus mean QALY for UC).

Probabilistic base case analysis

© 2025 Malfliet A et al. *JAMA Network Open*.

Probabilistic analysis was performed using non-parametric bootstrapping (n=1,000). The mean and bootstrapped standard error for total costs and QALYs were subsequently used to generate 1,000 iterations. Incremental costs and QALYs were presented in cost-effectiveness planes and the probability of the ICER being in the expected quadrant was calculated.

Scenario analyses

Next to the probabilistic base case analysis, 3 scenario analyses were performed to evaluate the impact of methodological decisions on the results of this CUA.

First, a scenario analysis included the cases with extreme cost values which were excluded for the base case analysis. Second, a complete case analysis was performed in which all cases with missing data for total healthcare costs or utilities on one of the follow-up moments were omitted from the analysis. The third scenario analysis comprised a CUA from a healthcare payer perspective from which the costs due to productivity loss were excluded.

eTable 3. Unit prices for Medication Use During Follow-Up

Substance	Dosage	Drug used for valuation[1]	Price (USD)/unit[2]
Acetylsalicylzuur	80 mg	Orifarm	0.063
Alprazolam	0.5 mg	Alprazolam teva	0.219
Amitriptyline	10 mg	Redomex	0.076
	25 mg		0.092
Atorvastatin	20 mg	Atorvastatin AB	0.259
Baclofen	10 mg	Baclofen Mylan	0.175
Bilastine	20 mg	Bellozal	0.366
Bisoprolol	2.5/6.25 mg	Co-Bisoprolol EG 98	0.152
Budesonide/Formoterol, fumarate	160 µg/4.5 µg	Symbicort	0.239
Buprenorphine	52.5 µg	Transtec transdermal patch	5.34
Bupropion	150 mg	Bupropion Teva	0.409

© 2025 Malfliet A et al. *JAMA Network Open*.

Butylhyoscine	10 mg	PIP Buscopan	0.277
Calcium/Cholecalciferol	1000 mg/1000 IU	D-Cure Calcium	0.476
	500 mg/200 IU	Steovit Orifarm Healthcare	0.234
Citalopram	20 mg	Citalopram Mylan	0.308
Clonazepam	0.5 mg	Rivotril Eurocept	0.093
Denosumab	120 mg	XGeva injection	334.583
Desloratadine	5 mg	Desloratadine PI-Pharma	0.175
Desogestrel	75 µg	Nacrez	0.199
Diazepam	5 mg	Diazepam Teva	0.182
DIclofenac	50 mg	Diclofenac Sandoz	0.190
	75 mg	Motifene	0.209
	75 mg	Diclofenac Apotex Retard	0.201
	2%	Voltaren Emul Gel	27.272
Domperidone	10 mg	Domperidon Teva	0.241
Duloxetine (hydrochloride)	30 mg	Duloxetin AB	0.308
Enoxaparin	2000 IU / 0.2	Ghemaxan	1.987
Escitalopram	10 mg	Escitalopram Teva	0.249
Esomeprazole	20 mg	Esomeprazole AB	0.197
Estradiol	1 mg	Progynova	0.116
Estradiol/Dydrogesterone	0.5 mg/2.5 mg	Femoston Impexeco	0.574
Ethinylestradiol/Drospirenone	0.02 mg/3 mg	Yadere	0.214
Folic acid	4 mg	Folavit	0.216
Fulvestrant	250 mg	Fulvestrant Accord injection	107.486
Gabapentine	300 mg	Gabapentine Sandoz	0.201

© 2025 Malfliet A et al. *JAMA Network Open*.

Glucosamine	1.178 mg	Dolenio	0.387
Ibuprofen	400 mg	Ibuprofen AB	0.093
	600 mg		0.150
Levocetirizine	5 mg	Levocetirizine AB	0.157
Levothyroxine	25 µg	L-Thyroxine	0.070
Lorazepam	1 mg	Lorazepam EG	0.094
	2.5 mg		0.163
Magnesium	162 mg	Ultra-MG	0.405
Mebeverine	200 mg	Duspatalin Retard	0.442
Melatonin	3 mg	Noxarem Melatonine	0.754
Melitracen/flupentixol	10 mg/0.5 mg	Deanxit	0.250
Meloxicam	15 mg	Meloxicam Teva	0.359
Methotrexate	2.5 mg	Ledertrexate Pfizer	0.332
Methylprednisolone	4 mg	Medrol Pfizer	0.286
Mometasone	50 µgram	Momepax spray	0.067
Montelukast	10 mg	Montelukast AB	0.567
Mucopolysaccharide, polysulphate	3 mg/g	Hirudoid cream	12.050
Naproxen	250 mg	Naproxen AB	0.191
Sodium hyaluronate/ectoine	0.5 mg/ml / 20 mg/ml	Hylo dual eye drops	0.068
Nitrofurantoin	50 mg	Furadantine MC	0.180
Olmesartan, medoxomil/Amlodipine	20 mg/5 mg	Forzaten filmomh tabl	0.426
Omeprazole	10 mg	Omeprazol AB	0.196
	20 mg		0.254
Otilonium, bromide	40 mg	Spasmomen	0.299
Oxycodone, hydrochloride	10 mg	Oxycodone Teva	0.227
Palbociclib	75 mg	Ibrance	186.330

© 2025 Malfliet A et al. *JAMA Network Open*.

Pantoprazole	20 mg	Pantoprazol Aurobindo	0.173
	40 mg	Pantoprazole EG	0.305
Paracetamol	500 mg	Paracetamol AB	0.083
	1000 mg	Algostase mono	0.099
Paracetamol/acetylsalicylic acid/coffeine	250 mg/250 mg/65 mg	Excedryn	0.363
Paracetamol/Codeine	500 mg/30 mg	Algocod	0.225
Paracetamol/Pseudo-ephedrine, hydrochloride	500 mg/30 mg	Parasineg	0.497
Paroxetine	20 mg	Paroxetine AB	0.310
Perindopril, arginine/Amlodipine	10/5 mg	Coveram	0.540
Perindopril, arginine/Indapamide	5/1.25 mg	Coversyl Plus	0.332
Pravastatin	20 mg	Pravastatine Apotex	0.198
Pregabalin	75 mg	Pregabaline Mylan	0.207
Propranolol	160 mg	Propranolol EG	0.242
Rosuvastatin	10 mg	Rosuvastatine EG	0.247
Salbutamol	100 µg	Ventolin inhaler	0.039
Salmeterol/flutiscasone	25 µgram/50 µgram	Seretide GSK	0.214
Sulfasalazine	500 mg	Salazopyrine Pfizer	0.115
Sumatriptan	10 mg	Imitrex	8.489
Thiamazole	10 mg	Strumazol	0.108
Tilidine/Naloxon	50 mg/4 mg	Valtran Retard	0.255
		Valtran drops	0.010
Topiramate	25 mg	Topiramate EG	0.211
Tramadol, hydrochloride	50 mg	Tramadol KRKA	0.177
	100 mg	Tramadol KRKA Retard	0.231

© 2025 Malfliet A et al. *JAMA Network Open*.

Tramadol/Paracetamol	37.5/325 mg	Tramadol/Paracetamol SMB	0.174
Trazodone, hydrochloride	100 mg	Trazodone EG tabl	0.127
Venlafaxine	37.5 mg	Efexor Exel	0.308
	75 mg	Venlafaxine Mylan Retard	0.272
Zolpidem	10 mg	Zolpidem Teva	0.392

[1]Brand names for the Belgian Pharmacy market.
[2]Unit prices comprise the price per unit of medication based on the least expensive package. If it was impossible to determine an individual unit of a certain medication (e.g., dermal creams and nasal sprays) the least expensive package price was used.
Abbreviations: mg: milligram(s); ml; millilitre(s); g: gram(s); μg: microgram(s); IU: international unit

© 2025 Malfliet A et al. *JAMA Network Open*.

eTable 4. Unit Prices for Visits With Clinicians

Healthcare provider	NIHDI Code[1]	Unit Price 2022 (USD)[2]
Medical Doctors		
Anaesthesiologist	105475	30.98
Cardiologist	102594	46.44
Dentist	301011	30.69
Dermatologist	102756	39.70
Endocrinologist	102874	71.85
Gastroenterologist	102616	46.44
General practitioner	101076	30.98
Gynaecologist	105593	30.98
Internal medicine	102550	54.83
Nephrologist	101614	64.57
Neurologist	102675	70.70
Occupational health physician	102535	30.98
Oncologist	102292	71.85
Ophthalmologist	102535	30.98
Orthopaedic surgeon	102535	30.98
Otorhinolaryngologist	105394	34.24
Physiatrist	105431	34.24
Plastic surgeon	105556	30.98
Pneumologist	102631	51.30
Psychiatrist	102690	58.32
Radiologist	105910	30.98
Rheumatologist	102653	69.63
Stomatologist	105711	30.98

© 2025 Malfliet A et al. *JAMA Network Open*.

Urologist	105630	30.98
Vascular surgeon	105711	34.24
Other specialists	102535	30.44
Other providers		
Dietician[3]		56.84
Homeopath[3]		56.84
Nurse	425014	4.83
Occupational therapist	794743	25.40
Physical therapist/osteopath/manual therapist[4]	567011	25.31
Psychologist	792654	85.93
Speech therapist	711314	33.29

[1]NIHDI nomenclature codes (explanation and unit prices available on the Nomensoft website: https://www.riziv.fgov.be/nl/toepassingen/Paginas/NomenSoft.aspx); [2]Unit prices are total prices, including the co-payment of the patient and the part reimbursed by the health insurance; [3]When no NIHDI nomenclature codes and unit prices were available; the internet was searched for reference prices in Belgium for the respective consultation; [4]The data did not allow for a subdivision among these practitioners. The cost is expected to be an underestimation because osteopathy is often more expensive as compared to physiotherapy under the NIHDI convention.
NIHDI: National Institute for Health and Disability Insurance (Belgium)

1.2. Detailed results of cost-utility analysis

Participant flow and characteristics

Data of 88 participants were available for the cost-utility analysis (CUA) (MPNA n=42; UC n=46). From the original RCT sample, 21 dropouts (did not complete the intervention) and 11 participants for whom no follow-up healthcare use (HCU) data were available (lost to follow up at T2 6-months follow-up), were excluded from the CUA. Baseline characteristics of the sample are presented in Table 1 and 2 in the main manuscript.

Costs

Intervention costs

© 2025 Malfliet A et al. *JAMA Network Open*.

The intervention incurred the same costs in both comparator arms (i.e., USD 532 per participant) representing an incremental cost equal to zero, wherefore this cost was omitted from the CUA.

Healthcare and productivity loss costs

Descriptives for healthcare and productivity loss costs are reported in detail in eTable5. Mean healthcare costs during 12 months follow-up were USD 972.58 (95% CI: USD 677.78; 1,267.38) in the MPNA group and USD 1,377.31 (95% CI: USD 912.49; 1,842.13) for the UC group, and were mainly determined by visits with healthcare providers, incurring a mean cost of USD 959.91 (95% CI: USD 628.45; 1,291.38) and USD 1,093.60 (95% CI: 743.40; 1,443.81), for MPNA and UC, respectively. Overall, the highest costs during follow-up were due to productivity loss with a mean cost of USD 3,758.33 (95% CI: USD 759.20; 6,653.58) in the MPNA group and USD 7,549.34 (95% CI: USD 897.09; 14,201.60) in the UC group. In terms of total costs, the sample included 7 cases with extreme values, which were excluded from the base case CUA, resulting in a mean total cost of USD 2,643.78 (95% CI: USD 1,828.46; 3,459.09) and USD 3,497.79 (95% CI: USD 2,395.89; 4,599.69) in the MPNA and UC group, respectively, considered for the base case CUA.

Utilities and QALYs

A small increase in utilities was seen from baseline to follow-up in both comparator groups. Mean utilities increased from 0.614 (95% CI: 0.586; 0.642) at baseline to 0.678 (95% CI: 0.647; 0.710) at 12 months follow-up in the MPNA group and from 0.619 (95% CI: 0.591; 0.647) to 0.659 (95% CI: 0.631; 0.686) in the UC group. After exclusion of cases presenting with extreme cost values (base case CUA), the mean QALY per participant was 0.891 (95% CI: 0.853; 0.929) for the MPNA group and 0.855 (95% CI: 0.824; 0.887) for the UC group. Detailed descriptives for utilities and QALYs are presented in eTable5.

eTable 5. Descriptives of Costs and effects for the MPNA and UC Groups

Costs (USD)	EXP				CON			
	Including cases with extreme cost values (n=42)		***Excluding cases with extreme cost values (n=39)[1]***		***Including cases with extreme cost values (n=46)***		***Excluding cases with extreme cost values (n=42)[1]***	
	Mean (95% CI)	*Median*	*Mean (95% CI)*	*Median*	*Mean (95% CI)*	*Median*	*Mean (95% CI)*	*Median*
Intervention[2]	532.03	532.03	532.03	532.03	532.03	532.03	532.03	532.03

© 2025 Malfliet A et al. *JAMA Network Open*.

Total HCU[3]	972.58 (677.78; 1,267.38)	650.93	957.35 (647.06; 1,267.65)	638.95	1,377.31 (912.49; 1,842.13)	1,016.98	1,403.87 (900.34; 1,907.39)	1,016.98
Healthcare visits[4]	959.91 (628.45; 1,291.38)	594.28	-	-	1,093.60 (743.40; 1,443.81)	650.26	-	-
Medication use[4]	78.70 (29.92; 127.47)	8.79	-	-	88.67 (42.07; 135.27)	19.87	-	-
Productivity loss[5]	3,758.33 (863.07; 969.48)	563.75	1,686.43 (934.96; 2,437.90)	375.83	7,549.34 (897.09; 14,201.60)	751.67	2,093.92 (1,023.91; 3,163.94)	375.83
**Total costs**	**4,730.91 (1,772.78; 7,689.04)**	**2,193.80**	**2,643.78 (1,828.46; 3,459.09)**	**1,700.19**	**8,926.65 (2,309.15; 15.544.10)**	**2,677.47**	**3,497.79 (2,395.89; 4,599.69)**	**2,464.06**
**Effects - utilities**								
Baseline	0.614 (0.586; 0.642)	0.608	0.610 (0.581; 0.640)	0.605	0.619 (0.591; 0.647)	0.616	0.615 (0.585; 0.646)	0.615
Post-intervention	0.691 (0.661; 0.722)	0.691	0.696 (0.663; 0.728)	0.696	0.665 (0.639; 0.691)	0.675	0.665 (0.637; 0.692)	0.675
6 months FU	0.680 (0.642; 0.718)	0.696	0.689 (0.650; 0.728)	0.700	0.659 (0.630; 0.689)	0.650	0.657 (0.627; 0.686)	0.650
12 months FU	0.678 (0.647; 0.710)	0.676	0.685 (0.653; 0.718)	0.676	0.659 (0.631; 0.686)	0.643	0.656 (0.627; 0.685)	0.643
**QALYs**	**0.883 (0.847; 0.919)**	**0.890**	**0.891 (0.853; 0.929)**	**0.920**	**0.858 (0.828; 0.888)**	**0.865**	**0.855 (0.824; 0.887)**	**0.867**

[1]Data used for the base case cost-utility analysis.
[2]Fixed cost related to the intervention comprising of 18 standard physiotherapy visits (identical for EXP and CON group) at a rate of USD 29.56 per visit.
[3]Costs related to HCU (healthcare visits and medication use) during the 12 month follow-up period.
[4]For the costs related to healthcare visits and medication use, specifically, data of only 77 cases (EXP: n=36; CON: n=41) was available since imputation occurred on the level of total healthcare costs.
[5]Costs related to productivity loss calculated by using the average labour cost of USD 49.5/hour for Belgium (i.e., Human Capital Approach).
Abbreviations: EXP: experimental intervention; CON: control intervention); n: sample size; 95% CI: 95% confidence interval; HCU: healthcare use; FU: follow-up; QALY: quality-adjusted life years

© 2025 Malfliet A et al. *JAMA Network Open*.

Base case probabilistic cost-utility analysis

For the base case, cases presenting with extreme values in terms of costs (n=7) were excluded from the analysis (base case CUA performed based on data of 81 cases). Mean costs and QALYs were USD 2,643.78 (bootstrapped 95% CI: USD 1,865.69; 3,468.84 - bootstrapped standard error (SE): 407.70) and 0.891 (bootstrapped 95%CI: 0.855; 0.928 - bootstrapped SE: 0.018) and USD 3,497.79 (bootstrapped 95%CI: USD 2,507.59; 4,562.65 - bootstrapped SE: USD 543.15) and 0.855 (bootstrapped 95%CI: 0.824; 0.884 - bootstrapped SE: 0.015) for MPNA and UC, respectively, resulting in an incremental cost of USD -854.01 and incremental QALY of 0.035. Probabilistic analysis showed 84.2% certainty for the ICER to be situated in the South-East quadrant of the cost-effectiveness plane (eFigure1).

eFigure 1. Cost-Effectiveness Plane of the Probabilistic Base Case Cost-Utility Analysis

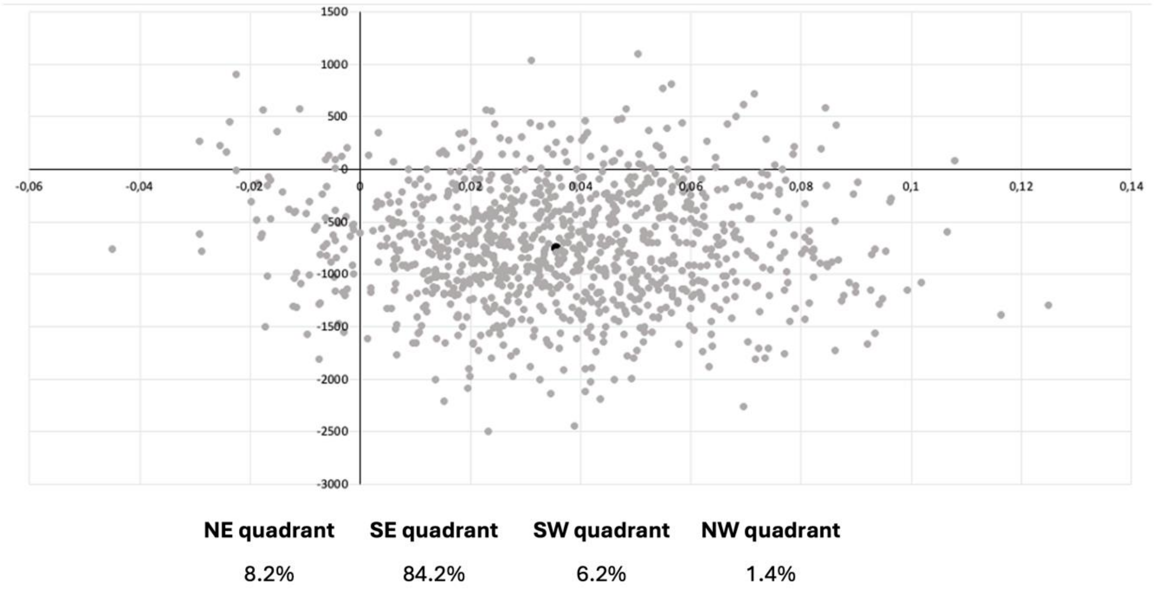

eFigure1. Cost-effectiveness plane of the probabilistic base case cost-utility analysis presenting the incremental costs (y-axis) and QALY (x-axis) for 1,000 iterations and the point estimate of the incremental cost-effectiveness ratio (ICER; black dot).

Abbreviations: NE: North-East; SE: South-East; SW: South-West; NW: North-West; QALY: Quality-Adjusted Life Years

© 2025 Malfliet A et al. *JAMA Network Open*.

Scenario analyses

For the first scenario analysis, the base case analysis was repeated with inclusion of the cases presenting extreme cost values. This resulted in mean costs of USD 4,730.91 (bootstrapped 95%CI: USD 2,515.71; 7,895.08 - bootstrapped SE: USD 1,396.63) and USD 8,926.65 (bootstrapped 95%CI: USD 3,781.19; 16,110.80 - bootstrapped SE: 3,277.21) for the MPNA and UC group, respectively, which led to a mean incremental cost of USD -4,195.73. Most of the iterations remained in the South-East quadrant of the cost-effectiveness plane, but the certainty for the ICER to be situated in that quadrant was lowered to 75.9 (eFigure2).

The third scenario analysis comprised a complete case version of the base case analysis excluding all cases with missing data for costs or utilities at one of the follow-up timepoints (n=15). The mean cost was USD 2,763.45 (bootstrapped 95% CI: 1,835.92; 3,671.04 – bootstrapped SE: 463.93) and USD 3,557.51 (bootstrapped 95% CI: 2,552.79; 4,830.96; bootstrapped SE: 603.54) for MPNA and UC, respectively. The mean QALY was 0.891 (bootstrapped 95% CI: 0.844; 0.935 – bootstrapped SE: 0.23) for the MPNA group and 0.862 (bootstrapped 95% CI: 0.827; 0.891 – bootstrapped SE: 0.016) for the UC group. The probabilistic analysis resulted in the same conclusion as the base case analysis but with higher uncertainty of the results (71.5% certainty for the ICER to be situated in the South-East quadrant) (eFigure3).

For the third scenario analysis a healthcare payer perspective was adopted, including only direct healthcare costs (hence, excluding costs related to productivity loss from the base case analysis). In the complete sample for CUA, 3 cases with extreme values for healthcare expenditure were identified and omitted from this scenario, resulting in a sample of n=85 (MPNA: n=41; UC: n=44). The mean costs for this scenario were USD 888.31 (bootstrapped 95% CI: USD 654.19; 1,158.21 – bootstrapped SE: 125.31) for the MPNA group and USD 1,107.11 (bootstrapped 95% CI: USD 872.99; 1,364.91 – bootstrapped SE: 126.26) for the UC group. Mean QALYs were equal to 0.890 (bootstrapped 95% CI: 0.854; 0.922 – bootstrapped SE: 0.017) and 0.863 (bootstrapped 95% CI: 0.833; 0.893 – bootstrapped SE: 0.015) for MPNA and UC, respectively. This resulted in an incremental cost of € -192.47 and QALY of 0.026. The probabilistic analysis showed 7838% certainty for the ICER to be situated in the South-East quadrant of the cost-effectiveness plane, hence for MPNA to dominate UC (eFigure4).

© 2025 Malfliet A et al. *JAMA Network Open*.

eFigure 2. Cost-Effectiveness Plane for Probabilistic Scenario Analysis No. 1

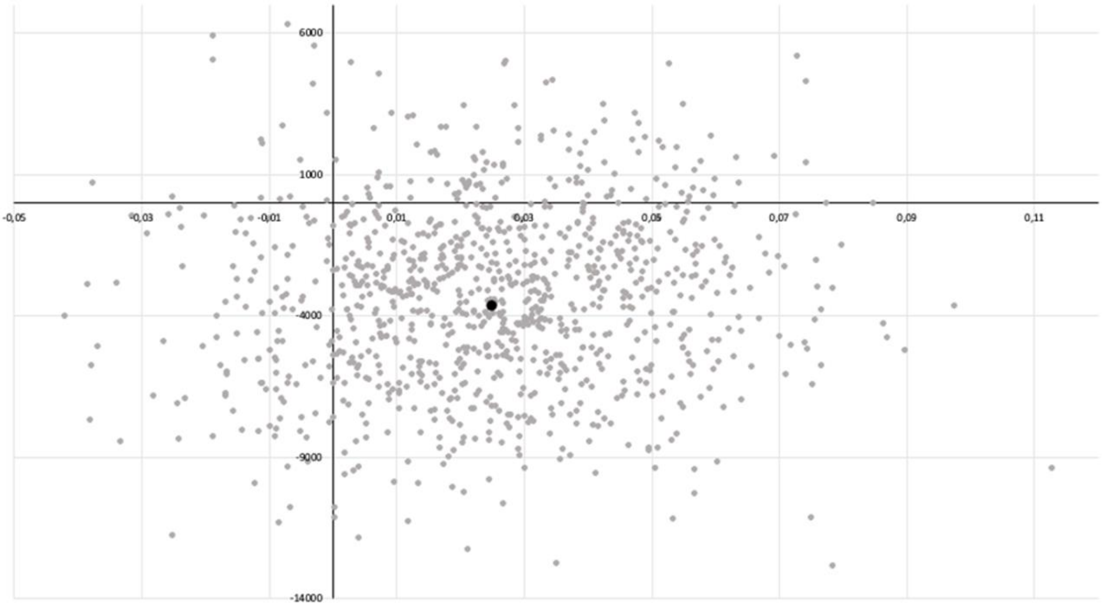

NE quadrant	SE quadrant	SW quadrant	NW quadrant
11.3%	75.9%	11.2%	1.6%

eFigure2. Cost-effectiveness plane for probabilistic scenario analysis no. 1 presenting the incremental costs (y-axis) and QALYs (x-axis) for 1,000 iterations and the point estimate of the incremental cost-effectiveness ratio (ICER; black dot). As compared to the base case analysis, for this scenario analysis cases presenting extreme cost values were included in the analysis.

Abbreviations: NE: North-East; SE: South-East; SW: South-West; NW: North-West; QALY: Quality-Adjusted Life Years

© 2025 Malfliet A et al. *JAMA Network Open*.

eFigure 3. Cost-Effectiveness Plane for Probabilistic Scenario Analysis No. 2

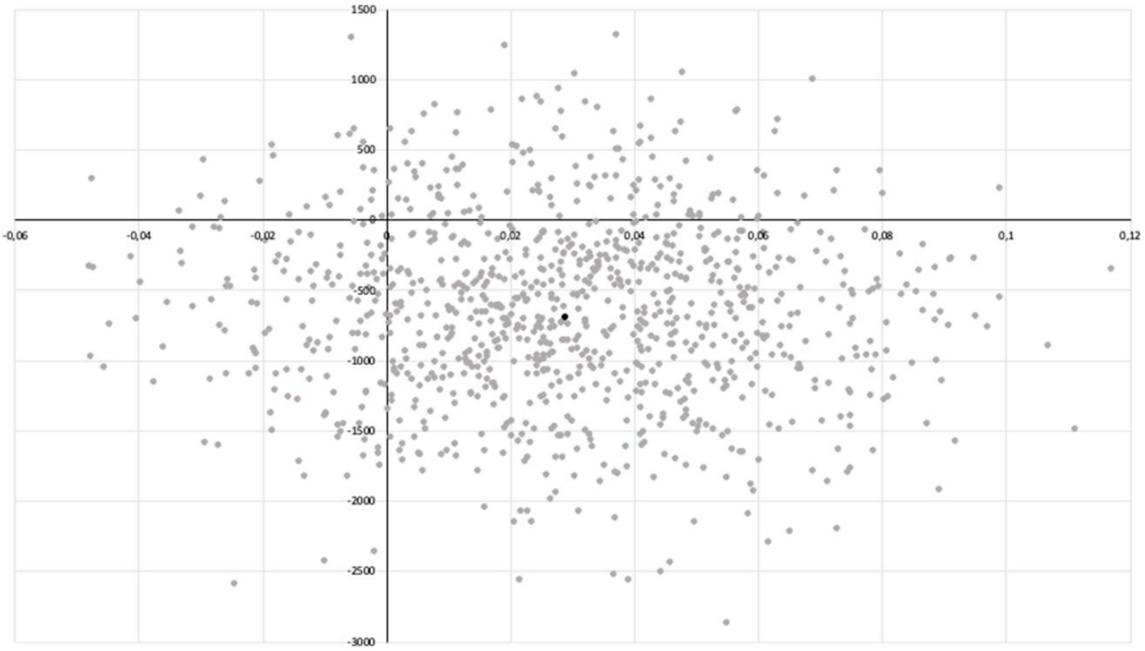

NE quadrant	SE quadrant	SW quadrant	NW quadrant
13.1%	71.5%	12.9%	2.5%

eFigure3. Cost-effectiveness plane for probabilistic scenario analysis no. 2 presenting the incremental costs (y-axis) and QALYs (x-axis) for 1,000 iterations and the point estimate of the incremental cost-effectiveness ratio (ICER; black dot). This scenario analysis comprises a complete case analysis of the base case analysis.

Abbreviations: NE: North-East; SE: South-East; SW: South-West; NW: North-West; QALY: Quality-Adjusted Life Years

© 2025 Malfliet A et al. *JAMA Network Open*.

eFigure 4. Cost-Effectiveness Plane for Probabilistic Scenario Analysis No. 3

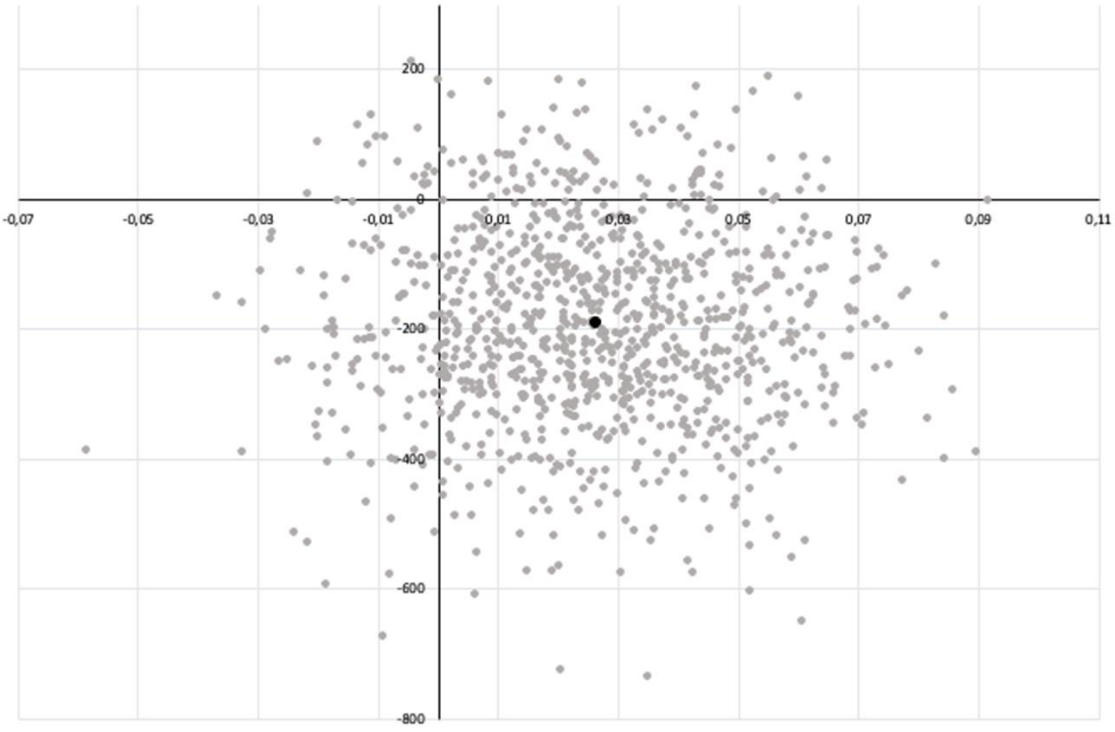

NE quadrant	SE quadrant	SW quadrant	NW quadrant
9.4%	78.8%	10%	1.8%

eFigure4. Cost-effectiveness plane for probabilistic scenario analysis no. 3 presenting the incremental costs (y-axis) and QALYs (x-axis) for 1,000 iterations and the point estimate of the incremental cost-effectiveness ratio (ICER; black dot). This scenario analysis represents the cost-utility analysis from a healthcare payer perspective, only including direct healthcare costs and excluding costs related to productivity loss. Abbreviations: NE: North-East; SE: South-East; SW: South-West; NW: North-West; QALY: Quality-Adjusted Life Years

Conclusion

MPNA dominated UC in both the base case and scenario analyses with a certainty between 72 and 84% for the ICER to be situated in the South-East quadrant. Based on the base case CUA, which applied a societal perspective, MPNA led to a mean cost saving of USD 854.01 and a small health gain of 0.035 QALY as compared to UC. When applying a healthcare payer perspective (scenario analysis 3), the mean cost saving of MPNA as compared to UC decreased to USD 218.80.

© 2025 Malfliet A et al. *JAMA Network Open*.

eTable 6. Overview of Missing Data per Outcome and Time Point

		MPNA intervention (n=60 included)				UC intervention (n=60 included)			
		Drop-out	Loss-to-follow-up	Variable missing data[1]	**Total missing**	Drop-out	Loss-to-follow-up	Variable missing data[1]	**Total missing**
EDPT	T1	13	-	1	**14**	8	-	1	**9**
	T2	13	6	3	**22**	8	5	6	**19**
TS	T1	13	-	0	**13**	8	-	0	**8**
	T2	13	6	1	**20**	8	5	3	**16**
CPM	T1	13	-	7	**20**	8	-	8	**16**
	T2	13	6	8	**27**	8	5	12	**25**
Quest	T1	13	-	1	**14**	8	-	2	**10**
	T2	13	6	0	**19**	8	5	0	**13**
	T3	13	6	0	**19**	8	5	0	**13**

EDPT: Electrical Detection and Pain Thresholds; TS: Temporal Summation; CPM: Conditioned Pain Modulation; Quest: Questionnaires. T1: immediately post-intervention; T2: 6 months post-intervention; T3: 12 months post-intervention (questionnaires only).

[1] The missing data in these columns is not due to dropouts or loss to follow-up. Instead, it represents cases where individuals have some variables missing at a specific time point, but they did complete other outcomes during that same time point.

© 2025 Malfliet A et al. *JAMA Network Open*.

eTable 7. Sensitivity Analyses, Corrected for Baseline NDI Levels

	Time-point	MPNA[a] (n=60) EMMean (SE)	UC[b] (n=60) EMMean (SE)	Mean group difference [95% CI]	Between-group *p-value[c] ; cohen(d)*
**NDI** (0-50)	T1	13.93 (0.82)	17.02 (0.82)	-3.09 [-5.27, -0.92]	P = 0.018; d = 1.015
	T2	15.27 (0.85)	17.19 (0.83)	-1.92 [-4.15, 0.31]	P = 0.273; d= 0.631
	T3	14.78 (0.87)	17.94 (0.86)	-3.16 [-5.46, -0.86]	P = 0.024; d = 1.036

[a]MPNA: Modern Pain Neuroscience Approach; [b]UC: Usual Care; [c]Bonferroni corrected

To assess the effect of treatment group over time on NDI scores, we fit a linear mixed-effects model with subject as random intercept using post-baseline data (i.e., excluding baseline measurement). The model included fixed effects for Group, Timepoint, and their interaction (Group*Timepoint), as well as covariates for baseline NDI value, age, and gender. We fitted the following model:

$$NDI_{ij} = \beta_0 + \beta_1 \cdot \mathrm{Group}_i + \beta_2 \cdot \mathrm{Timepoint}_j + \beta_3 \cdot \left(\mathrm{Group}_i \times \mathrm{Timepoint}_j\right) + \beta_4 \cdot \mathrm{NDI}_{\mathrm{baseline},i} + \beta_5 \cdot \mathrm{Age}_i + \beta_6 \cdot \mathrm{Sex}_i + u_i + \epsilon_{ij}$$

where $\mathrm{i} = 1, \dots, \mathrm{N}$ indexes subjects, and $\mathrm{j} = 2,3,4$ indexes post-baseline timepoints. $\mathrm{u_i} \sim \mathcal{N}(0, \sigma_u^2)$ is the random intercept for subject. $\epsilon_{ij} \sim \mathcal{N}(0, \sigma^2)$ is the residual error.

Estimated marginal means and pairwise between-groups differences at each timepoint were computed using the emmeans package. By including the outcome baseline value as a covariate, the model provides adjusted group comparisons over time, controlling for individual differences in baseline severity. This approach ensures a more accurate estimation of group effects over time and is consistent with recommendations for analyzing longitudinal data with baseline covariate adjustment.

eTable 8. Sensitivity Analysis, Baseline Characteristics of Completers vs Dropouts

	UC		MPNA	
	**Drop-out (N=18)**	**Completers (N=42)**	**Drop-out (N=25)**	**Completers (N=35)**
**Gender**				
Male	6 (33.3%)	7 (16.7%)	5 (20.8%)	13 (36.1%)
Female	12 (66.7%)	35 (83.3%)	19 (79.2%)	23 (63.9%)
**Age** (years)	39.3 (9.99)	43.7 (12.3)	41.1 (12.1)	40.1 (9.98)
**BMI**	26.2 (3.50)	24.6 (3.54)	24.7 (4.83)	24.9 (4.59)
**Academic education**				
Primary Education	2 (11.1%)	1 (2.4%)	0 (0%)	0 (0%)
Secondary Education	4 (22.2%)	15 (35.7%)	10 (41.7%)	15 (41.7%)
Vocational Education/Training	8 (44.4%)	18 (42.9%)	11 (45.8%)	14 (38.9%)
Bachelors/Master/PhD Degree	4 (22.2%)	8 (19.0%)	3 (12.5%)	7 (19.4%
**Marital Status**				
Single	6 (33.3%)	13 (31.0%)	6 (25.0%)	7 (19.4%)
Married	7 (38.9%)	15 (35.7%)	8 (33.3%)	15 (41.7%)
Cohabitation	3 (16.7%)	11 (26.2%)	4 (16.7%)	7 (19.4%)
Divorced	1 (5.6%)	3 (7.1%)	4 (16.7%)	6 (16.7%)
Widowed	1 (5.6%)	0 (0%)	2 (8.3%)	1 (2.8%)
**Employment Status**				
Student	0 (0%)	5 (11.9%)	0 (0%)	0 (0%)
Unemployed	1 (5.6%)	1 (2.4%)	1 (4.2%)	2 (5.6%)
Self-employed	0 (0%)	0 (0%)	2 (8.3%)	1 (2.8%)
Employed	12 (66.7%)	26 (61.9%)	17 (70.8%)	31 (86.1%)
Incapacitated	5 (27.8%)	7 (16.7%)	3 (12.5%)	1 (2.8%)
Retired	0 (0%)	3 (7.1%)	0 (0%)	1 (2.8%)
**Annual Income**				
< €10.000	0 (0%)	0 (0%)	1 (4.2%)	0 (0%)
€10.000-€20.000	2 (11.1%)	5 (11.9%)	5 (20.8%)	7 (19.4%)
€20.000-€40.000	11 (61.1%)	22 (52.4%)	9 (37.5%)	10 (27.8%)
€40.000-€60.000	3 (16.7%)	5 (11.9%)	6 (25.0%)	16 (44.4%)
> €60.000	1 (5.6%)	4 (9.5%)	2 (8.3%)	3 (8.3%)
**Insurance**				
No	4 (22.2%)	9 (21.4%)	10 (41.7%)	7 (19.4%)
Not anymore	4 (22.2%)	20 (47.6%)	10 (41.7%)	16 (44.4%)
Yes	10 (55.6%)	13 (31.0%)	4 (16.7%)	13 (36.1%)
**Litigation**				
No	16 (88.9%)	36 (85.7%)	24 (100%)	31 (86.1%)
Yes	2 (11.1%)	6 (14.3%)	0 (0%)	5 (13.9%)
**Compensation**				
No	9 (50.0%)	20 (47.6%)	10 (41.7%)	16 (44.4%)
Yes	3 (16.7%)	9 (21.4%)	10 (41.7%)	10 (27.8%)
Pending	6 (33.3%)	13 (31.0%)	4 (16.7%)	10 (27.8%)
**Previous whiplash injury**				
No	12 (66.7%)	32 (76.2%)	17 (70.8%)	21 (58.3%)
Yes	6 (33.3%)	10 (23.8%)	7 (29.2%)	15 (41.7%)
**Medication intake**				
No	10 (55.6%)	23 (54.8%)	16 (66.7%)	18 (50.0%)
Yes	8 (44.4%)	19 (45.2%)	8 (33.3%)	18 (50.0%)
**Days with pain/week**	5.83 (1.86)	5.76 (1.51)	5.42 (1.74)	6.22 (1.22)
**Pain distribution**	3.30 (2.45)	3.54 (3.84)	2.49 (1.64)	3.56 (3.31)
**Average pain previous week** NPRS (0-10)	5.78 (1.59)	5.19 (1.76)	5.42 (1.86)	5.28 (1.68)
**Physical functioning** PSC (0-30)	20.1 (6.75)	19.2 (4.83)	20.0 (4.49)	19.8 (4.82)
**Neck-related disability** NDI (0-50)	20.8 (4.40)	19.5 (5.41)	19.3 (5.20)	18.1 (4.64)
**Pain catastrophizing** PCS (0-52)	25.7 (10.2)	24.0 (11.2)	22.5 (13.3)	24.3 (10.7)
**Pain-related fear** PASS-20 (0-100)	44.5 (22.1)	32.1 (16.3)	33.5 (23.3)	30.4 (15.7)
**CS-related distress CSI (0-100)**	56.3 (11.2)	45.9 (13.6)	45.7 (11.5)	43.7 (13.4)
**Post-traumatic stress** IES (0-88)[†]	20.0 [3.00, 59.0]	16.0 [4.00, 65.0]	16.5 [1.00, 61.0]	7.50 [0, 51.0]
**Illness perception** IPQ (0-204)	132 (13.2)	129 (12.2)	129 (11.3)	127 (14.1)

© 2025 Malfliet A et al. *JAMA Network Open*.

eReferences.

1. Goudman L, Huysmans E, Coppieters I, et al. Electrical (Pain) Thresholds and Conditioned Pain Modulation in Patients with Low Back-Related Leg Pain and Patients with Failed Back Surgery Syndrome: A Cross-Sectional Pilot Study. *Pain Med*. Mar 1 2020;21(3):538-547. doi:10.1093/pm/pnz118
2. Cathcart S, Winefield AH, Rolan P, Lushington K. Reliability of temporal summation and diffuse noxious inhibitory control. *Pain research & management : the journal of the Canadian Pain Society = journal de la societe canadienne pour le traitement de la douleur*. 2009;14:433-438.
3. Price DD, Staud R, Robinson ME, Mauderli AP, Cannon R, Vierck CJ. Enhanced temporal summation of second pain and its central modulation in fibromyalgia patients. *Pain*. Sep 2002;99(1-2):49-59. doi:10.1016/s0304-3959(02)00053-2
4. Lewis GN, Rice DA, McNair PJ. Conditioned pain modulation in populations with chronic pain: a systematic review and meta-analysis. *J Pain*. Oct 2012;13(10):936-44. doi:10.1016/j.jpain.2012.07.005
5. Motohashi K, Umino M. Heterotopic painful stimulation decreases the late component of somatosensory evoked potentials induced by electrical tooth stimulation. *Brain Res Cogn Brain Res*. Mar 2001;11(1):39-46. doi:10.1016/s0926-6410(00)00062-8
6. Terkelsen AJ, Andersen OK, Hansen PO, Jensen TS. Effects of heterotopic- and segmental counter-stimulation on the nociceptive withdrawal reflex in humans. *Acta Physiol Scand*. Jul 2001;172(3):211-7. doi:10.1046/j.1365-201x.2001.00856.x
7. Savic G, Bergström EM, Frankel HL, Jamous MA, Ellaway PH, Davey NJ. Perceptual threshold to cutaneous electrical stimulation in patients with spinal cord injury. *Spinal Cord*. Sep 2006;44(9):560-6. doi:10.1038/sj.sc.3101921
8. Nickel FT, DeCol R, Jud S, Fasching PA, Seifert F, Maihöfner C. Inhibition of hyperalgesia by conditioning electrical stimulation in a human pain model. *Pain*. Jun 2011;152(6):1298-1303. doi:10.1016/j.pain.2011.02.005
9. Vuilleumier PH, Biurrun Manresa JA, Ghamri Y, et al. Reliability of Quantitative Sensory Tests in a Low Back Pain Population. *Reg Anesth Pain Med*. Nov-Dec 2015;40(6):665-73. doi:10.1097/aap.0000000000000289
10. Banic B, Petersen-Felix S, Andersen OK, et al. Evidence for spinal cord hypersensitivity in chronic pain after whiplash injury and in fibromyalgia. *Pain*. 2004;107:7-15.
11. Goudman L, Laton J, Brouns R, et al. Cortical mapping of painful electrical stimulation by quantitative electroencephalography: unraveling the time-frequency-channel domain. *J Pain Res*. 2017;10:2675-2685. doi:10.2147/jpr.S145783
12. Biurrun Manresa JA, Neziri AY, Curatolo M, Arendt-Nielsen L, Andersen OK. Test-retest reliability of the nociceptive withdrawal reflex and electrical pain thresholds after single and repeated stimulation in patients with chronic low back pain. *Eur J Appl Physiol*. Jan 2011;111(1):83-92. doi:10.1007/s00421-010-1634-0
13. Yarnitsky D. Role of endogenous pain modulation in chronic pain mechanisms and treatment. *Pain*. Apr 2015;156 Suppl 1:S24-s31. doi:10.1097/01.j.pain.0000460343.46847.58

© 2025 Malfliet A et al. *JAMA Network Open*.

14. Yarnitsky D, Bouhassira D, Drewes AM, et al. Recommendations on practice of conditioned pain modulation (CPM) testing. *Eur J Pain*. Jul 2015;19(6):805-6. doi:10.1002/ejp.605
15. Moont R, Pud D, Sprecher E, Sharvit G, Yarnitsky D. 'Pain inhibits pain' mechanisms: Is pain modulation simply due to distraction? *Pain*. Jul 2010;150(1):113-120. doi:10.1016/j.pain.2010.04.009
16. Kennedy DL, Kemp HI, Ridout D, Yarnitsky D, Rice ASC. Reliability of conditioned pain modulation: a systematic review. *Pain*. Nov 2016;157(11):2410-2419. doi:10.1097/j.pain.0000000000000689
17. Fujii-Abe K, Oono Y, Motohashi K, Fukayama H, Umino M. Heterotopic CO2 laser stimulation inhibits tooth-related somatosensory evoked potentials. *Pain Med*. Jun 2010;11(6):825-33. doi:10.1111/j.1526-4637.2010.00855.x
18. Piché M, Watanabe N, Sakata M, et al. Basal μ-opioid receptor availability in the amygdala predicts the inhibition of pain-related brain activity during heterotopic noxious counter-stimulation. *Neurosci Res*. Apr-May 2014;81-82:78-84. doi:10.1016/j.neures.2014.02.006
19. Cleemput I, Neyt M, Van de Sande S, Thiry N. *Belgische richtlijnen voor economische evaluaties en budget impact analyses: tweede editie. Health Technology Assessment (HTA)*. 2012.
20. EUnetHTA. *Methods for health economic evaluations – A guideline based on current practices in Europe*. 2015.
21. Drummond MF, Sculpher MJ, Claxton K, Stoddart GL, Torrance GW. *Methods for the Economic Evaluation of Health Care Programmes - Fourth Edition*. 4 ed. Oxford University Press; 2015.
22. Husereau D, Drummond M, Augustovski F, et al. Consolidated Health Economic Evaluation Reporting Standards 2022 (CHEERS 2022) Statement: Updated Reporting Guidance for Health Economic Evaluations. *Value Health*. Jan 2022;25(1):3-9. doi:10.1016/j.jval.2021.11.1351
23. cijfers S-Bi. Gezondheidsindex. Accessed March 26, 2021, 2021. https://statbel.fgov.be/nl/themas/consumptieprijsindex/gezondheidsindex
24. van den Brink M, van den Hout WB, Stiggelbout AM, Putter H, van de Velde CJ, Kievit J. Self-reports of health-care utilization: diary or questionnaire? *International journal of technology assessment in health care*. Summer 2005;21(3):298-304.
25. Severs M, Petersen RE, Siersema PD, Mangen MJ, Oldenburg B. Self-reported Health Care Utilization of Patients with Inflammatory Bowel Disease Correlates Perfectly with Medical Records. *Inflammatory bowel diseases*. Mar 2016;22(3):688-93. doi:10.1097/mib.0000000000000643
26. (BCFI/CBIP) BCVFI. *Gecommentarieerd Geneesmiddelenrepertorium*. BCFI/CBIP; 2020.
27. (NIHDI/RIZIV-INAMI) NIfHaDI. Nomensoft. National Institute for Health and Disability Insurance. https://ondpanon.riziv.fgov.be/Nomen/nl/search
28. Aaronson NK, Muller M, Cohen PD, et al. Translation, validation, and norming of the Dutch language version of the SF-36 Health Survey in community and chronic disease populations. *J Clin Epidemiol*. Nov 1998;51(11):1055-68. doi:10.1016/s0895-4356(98)00097-3
29. Kennedy-Martin M, Slaap B, Herdman M, et al. Which multi-attribute utility instruments are recommended for use in cost-utility analysis? A review of national health

© 2025 Malfliet A et al. *JAMA Network Open*.

technology assessment (HTA) guidelines. *Eur J Health Econ*. Nov 2020;21(8):1245-1257. doi:10.1007/s10198-020-01195-8
30. Brazier JE, Roberts J. The estimation of a preference-based measure of health from the SF-12. *Medical care*. Sep 2004;42(9):851-9. doi:10.1097/01.mlr.0000135827.18610.0d
31. Walters SJ, Brazier JE. What is the relationship between the minimally important difference and health state utility values? The case of the SF-6D. *Health Qual Life Outcomes*. Apr 11 2003;1:4. doi:10.1186/1477-7525-1-4

© 2025 Malfliet A et al. *JAMA Network Open*.
