# Supplementary material for: Pain Science Education, Stress Management, and Cognition-Targeted Exercise Therapy in Chronic Whiplash Disorders: A Randomized Clinical Trial
Source: JAMA Netw Open. 2025 Aug 12;8(8):e2526674. doi: 10.1001/jamanetworkopen.2025.26674 (PMC12344539; doi:10.1001/jamanetworkopen.2025.26674)
Supplement: Supplement 3. — Data Sharing Statement [file jamanetwopen-e2526674-s003.pdf]

Data Sharing Statement

Malfliet. Pain Science Education, Stress Management, and Cognition-Targeted Exercise Therapy in Chronic Whiplash Disorders. *JAMA Netw Open*. Published August 12, 2025. doi:10.1001/jamanetworkopen.2025.26674

Data
Additional Information: Trial Registration: ClinicalTrials.gov : identifier number NCT03239938
Data available: No

Additional Information
Explanation for why data not available: Given the sensitive nature of the data, public access to it is not possible.
